# Shoulder replacement surgery’s rising demand, inequality of provision, and variation in outcomes: cohort study using Hospital Episode Statistics for England

**DOI:** 10.1186/s12916-023-03112-1

**Published:** 2023-10-26

**Authors:** Epaminondas Markos Valsamis, Rafael Pinedo-Villanueva, Adrian Sayers, Gary S. Collins, Jonathan L. Rees

**Affiliations:** 1 Nuffield Department of Orthopaedics, Rheumatology and Musculoskeletal Sciences, Botnar Research Centre, University of Oxford, Oxford, OX3 7LD UK; 2 NIHR Oxford Biomedical Research Centre, Oxford, UK; 3 Musculoskeletal Research Unit, Bristol Medical School, Southmead Hospital, University of Bristol, Bristol, UK; 4 Nuffield Department of Orthopaedics, Rheumatology and Musculoskeletal Sciences, Centre for Statistics in Medicine, University of Oxford, Oxford, OX3 7LD UK

**Keywords:** Shoulder replacement, Temporal trends, Service provision, Cohort study

## Abstract

**Background:**

The aim of this study was to forecast future patient demand for shoulder replacement surgery in England and investigate any geographic and socioeconomic inequalities in service provision and patient outcomes.

**Methods:**

For this cohort study, all elective shoulder replacements carried out by NHS hospitals and NHS-funded care in England from 1999 to 2020 were identified using Hospital Episode Statistics data. Eligible patients were aged 18 years and older. Shoulder replacements for malignancy or acute trauma were excluded. Population estimates and projections were obtained from the Office for National Statistics. Standardised incidence rates and the risks of serious adverse events (SAEs) and revision surgery were calculated and stratified by geographical region, socioeconomic deprivation, sex, and age band. Hospital costs for each admission were calculated using Healthcare Resource Group codes and NHS Reference Costs based on the National Reimbursement System. Projected rates and hospital costs were predicted until the year 2050 for two scenarios of future growth.

**Results:**

A total of 77,613 elective primary and 5847 revision shoulder replacements were available for analysis. Between 1999 and 2020, the standardised incidence of primary shoulder replacements in England quadrupled from 2.6 to 10.4 per 100,000 population, increasing predominantly in patients aged over 65 years. As many as 1 in 6 patients needed to travel to a different region for their surgery indicating inequality of service provision. A temporal increase in SAEs was observed: the 30-day risk increased from 1.3 to 4.8% and the 90-day risk increased from 2.4 to 6.0%. Patients from the more deprived socioeconomic groups appeared to have a higher risk of SAEs and revision surgery. Shoulder replacements are forecast to increase by up to 234% by 2050 in England, reaching 20,912 procedures per year with an associated annual cost to hospitals of £235 million.

**Conclusions:**

This study reports a rising incidence of shoulder replacements, regional disparities in service provision, and an overall increasing risk of SAEs, especially in more deprived socioeconomic groups. These findings highlight the need for better healthcare planning to match local population demand, while more research is needed to understand and prevent the increase observed in SAEs.

**Supplementary Information:**

The online version contains supplementary material available at 10.1186/s12916-023-03112-1.

## Background

Shoulder pain not only leads to higher healthcare utilisation but also can curtail a patient’s working life expectancy by 1.8 to 8.1 years, depending on their age [1–3]. Most shoulder problems are related to degenerative and inflammatory joint disorders, and shoulder replacements are an effective surgical treatment for managing pain and improving function in patients with end-stage joint arthritis. Despite the global increase in shoulder replacements, the growth rate varies across countries, and the United Kingdom (UK) has been reported to exhibit lower rates [4–6]. However, literature regarding incidence rates for shoulder replacements and access to care in the UK remains scarce. Accurate estimates of the trends in shoulder replacement surgery, access across patient groups, and growth forecasts are vital for ensuring adequate resource planning and timely healthcare provision for all patients suffering from pain and disability.

The COVID-19 pandemic has exacerbated the already rising waiting times for elective surgery worldwide. In the UK, there has been much discussion around service provision changes to address these waiting times [7]. A key prerequisite to the National Health Service’s (NHS) delivery plan to manage the backlog includes a thorough understanding of performance variation across regions and levels of deprivation [8]. Addressing inequalities generated by variable access to care is crucial for enhancing overall health outcomes. In the context of shoulder replacement surgery, it is important to ensure consistency in services and patient outcomes after surgery. Therefore, a comprehensive understanding of any variations in healthcare access and postoperative outcomes across different geographic regions and patient groups is essential for evidence-based policymaking and practice [9].

The aim of this study was to investigate the changes in the incidence of elective shoulder replacement surgery in England and forecast its demand and the associated costs to the NHS over the next three decades. A further aim was to examine for any geographic and socioeconomic inequalities in service provision and patient outcomes.

## Methods

### Study design

This is a population-based cohort study using routinely collected Hospital Episode Statistics data in England from 1 January 1999 to 31 December 2020.

### Data sources

Records for all patients undergoing elective shoulder replacement surgery in England were available from the Hospital Episode Statistics (HES) Admitted Patient Care (APC) database managed by NHS Digital. The HES APC database provides universal coverage of all inpatient and day case activity carried out by NHS hospitals and NHS-funded care in England and contains demographic data, medical diagnoses, and procedural and administrative information. Data submission from hospital providers is mandatory to ensure accurate reimbursement for all activity performed. Data were linked to the Civil Registration Mortality database. Population estimates by age, sex, and year within each Government Office Region (GOR) were obtained from the Office for National Statistics (ONS) and linked to the HES data for analysis [10]. National population projections per 5-year age groups and sex were obtained from the ONS for the years 2021 through 2050 [11].

The study dataset consisted of all episodes for included patients, linkable by a valid pseudonymised patient identifier. The index operative episode was identified as the first episode containing a procedure for a shoulder replacement per side. Subsequent shoulder replacement procedures on the same side were identified as repeat (revision) surgery. Revisions included in this study were restricted to those linked to elective primary procedures that met the eligibility criteria. The three types of shoulder replacement procedures (humeral hemiarthroplasty [HA], conventional total shoulder replacement [TSR], and reverse total shoulder replacement [RTSR]), as well as revisions, were identified from combinations of primary/revision and anatomy OPCS-4 codes (see Additional file 1). The GOR of residence for each shoulder replacement procedure was identified from the patient’s outward code (first part of the postcode). While GORs closed in 2011, this regional geography is maintained for statistical purposes and is referred to as ‘regions’. Patient socioeconomic status was assigned using the Index of Multiple Deprivation (IMD). This is a combined measure of deprivation capturing income, employment, education, health, crime, barriers to housing and services, and living environment domains [12]. IMD overall rankings were used to categorise patients into five IMD groups from the most deprived 20% to the least deprived 20%. Population data stratified by IMD fifths were only available from 2001 onwards (IMD areas were created in line with the 2001 Census).

Serious adverse events (SAEs) were defined as medical complications severe enough to require admission to hospital including pulmonary embolism, myocardial infarction, lower respiratory tract infection, acute kidney injury, urinary tract infection, cerebrovascular events, and all-cause death [9]. SAEs were identified using ICD-10 codes and categorised into those occurring within 30 or 90 days from the index procedure.

The NHS HRG4 + 2022/23 national costs grouper was used to generate Healthcare Resource Group (HRG) codes for each index operative spell [13]. Each operative spell may consist of one or more episodes, including inpatient activity before or after the operative episode, enabling the capture of all inpatient activity related to that index procedure. HRGs were valued using the 2020–2021 NHS Reference Costs to generate the reimbursement value of each procedure to the hospital provider based on the National Reimbursement System [14].

### Eligibility criteria

All patients aged 18 years and older who had an OPCS-4 code for a primary shoulder replacement were eligible for inclusion in the study. Patients were excluded if the main indication for surgery was acute trauma or malignancy, based on ICD-10 diagnostic codes. Patients were excluded if their surgical history was inconsistent (i.e. their date of revision or death predated their primary surgery) or contained duplicates.

### Patient and public involvement

Several of the top ten research uncertainties from the 2015 James Lind Alliance Priority Setting Partnership on shoulder surgery related to shoulder replacements [15]. A Patient Advisory Panel for this study highlighted the importance of equitable access to shoulder replacement services across the country to reduce travel for elective surgery. We therefore also planned to analyse the availability of surgical units providing shoulder replacements in each region.

### Statistical analyses

Descriptive statistics were used to summarise patient demographics. Population data from the ONS were used to calculate standardised incidence rates by year of treatment, stratified by region, IMD fifth, age band, and sex, following the methodology of the Association of Public Health Observatories, using direct age and sex standardisation [16]. Age- and sex-standardised risks were calculated for SAEs within 30 and 90 days of surgery. Risks for each type of SAE were also analysed separately. For revision surgery, we were interested in the net failure of the implant, and so the Kaplan–Meier estimator was used to estimate the risk of revision at 1, 3, 5, and 10 years following primary shoulder replacement. Flexible parametric survival models were used to estimate the age- and sex-adjusted risk of revision at each follow-up period as the proportional hazards assumption for these variables did not hold (precluding analysis using a simpler Cox model) [17].

Service provision for each region was evaluated by calculating the number of surgical units providing shoulder replacement surgery per 100,000 population for each region of treatment (surgical unit density) per year and comparing this to the regional incidence of elective primary shoulder replacement procedures. The rate of travel for treatment was calculated by comparing the region of patient residence to that of treatment (the hospital provider’s region).

Two different scenarios were considered to calculate projections for shoulder replacement surgery demand. Scenario 1 used an age- and sex-standardised incidence rate that was held constant at the 2019 levels (preceding the COVID-19 pandemic) while scenario 2 used a linear extrapolation of the age- and sex-standardised incidence rate for the study period up to 2019 [18]. For scenario 2, separate linear regression models were fit to historical data for each 5-year age band and sex cohort, using year of surgery as a covariate, and predictions derived for future years. Data from 2020 were not used for forecasting due to the marked effect of the COVID-19 pandemic on surgical volume. The corresponding incidence rates for each scenario were applied to national population projection data from the ONS to calculate the expected standardised incidence and hence absolute volume predictions for 2021 through 2050. The forecast cost was calculated by applying the 5-year age band and sex-stratified mean costs for 2019 to the surgical volume projections. All historic and forecast costs are presented in 2021 GBP, as all admissions were valued according to the latest available NHS Reference Costs (2020–2021) at the time of conducting the study.

Data for either region or IMD were missing for a total of 1029 patients (1.3% of the study dataset). No data was missing for any other variables included in this study. These records were excluded, and a complete case analysis was undertaken (see Additional file 1 for data flowchart and for baseline characteristics and outcomes of observations with missing data) [19, 20]. A total of 6% of procedures did not generate a valid HRG code, so historical costs only reflect 94% of shoulder replacement surgery undertaken. The geographic information system, QGIS V.3.82, was used to graphically summarise standardised incidence rates for each region in England, per year [21]. Study findings are reported in accordance with the REporting of studies Conducted using Observational Routinely-collected health Data (RECORD) recommendations (see Additional file 2) [22]. Stata V.16.1 (StataCorp) was used to perform all statistical analyses [23].

## Results

Between 1 January 1999 and 31 December 2020, a total of 77,613 elective primary and 5847 revision shoulder replacements were performed on 68,370 patients (Additional file 1: Fig. S1). The maximum follow-up for primary procedures was 22 years with 482,418 years of observation time. A summary of patient demographics is shown in Table 1. The average patient age at primary shoulder replacement steadily increased from 67.9 (SD 12.9) in 1999 to 72.4 (SD 9.6) years in 2019, while that of revision shoulder replacement remained more stable. Regional and socioeconomic trends in age are shown in Additional file 1.

**Table 1:** Elective primary shoulder replacement patient demographics by calendar year

Year	Procedures (*n*)	Patients (*n*)	Women		Age at primary		Age at revision	
			**(*n*)**	**(%)**	**Mean**	**SD**	**Mean**	**SD**
1999	1231	1218	908	73.8	67.9	12.9	69.5	12.2
2000	1295	1280	957	73.9	68.5	12.1	60.5	19.2
2001	1277	1267	940	73.6	69.1	12.1	68.9	10.1
2002	1428	1419	1061	74.3	69.1	12.3	66.1	13.3
2003	1779	1765	1302	73.2	69.2	12.2	67.8	12.0
2004	1921	1899	1437	74.8	69.8	11.3	68.6	12.5
2005	2326	2296	1722	74.0	69.8	11.3	68.7	10.8
2006	2307	2291	1633	70.8	69.7	11.4	68.0	11.9
2007	2823	2786	1992	70.6	69.4	11.7	69.8	10.9
2008	3373	3338	2432	72.1	70.4	10.9	69.9	10.8
2009	3578	3524	2550	71.3	70.6	11.1	68.1	11.2
2010	3615	3584	2576	71.3	71.0	11.1	70.3	10.5
2011	4016	3957	2834	70.6	70.9	10.8	68.9	12.5
2012	4355	4294	3060	70.3	71.2	10.7	69.2	10.4
2013	4679	4636	3272	69.9	71.2	10.6	68.2	11.2
2014	5296	5224	3772	71.2	71.9	10.0	69.3	11.2
2015	5222	5171	3682	70.5	71.6	10.3	69.2	11.1
2016	5680	5627	3957	69.7	71.8	10.3	69.9	10.8
2017	5925	5867	4106	69.3	72.2	9.8	69.7	11.7
2018	6121	6052	4232	69.1	72.3	9.8	69.6	10.7
2019	6268	6197	4349	69.4	72.4	9.6	70.3	11.3
2020	3098	3085	2100	67.8	72.2	9.9	70.1	10.1

### National trends

**Fig. 1:**
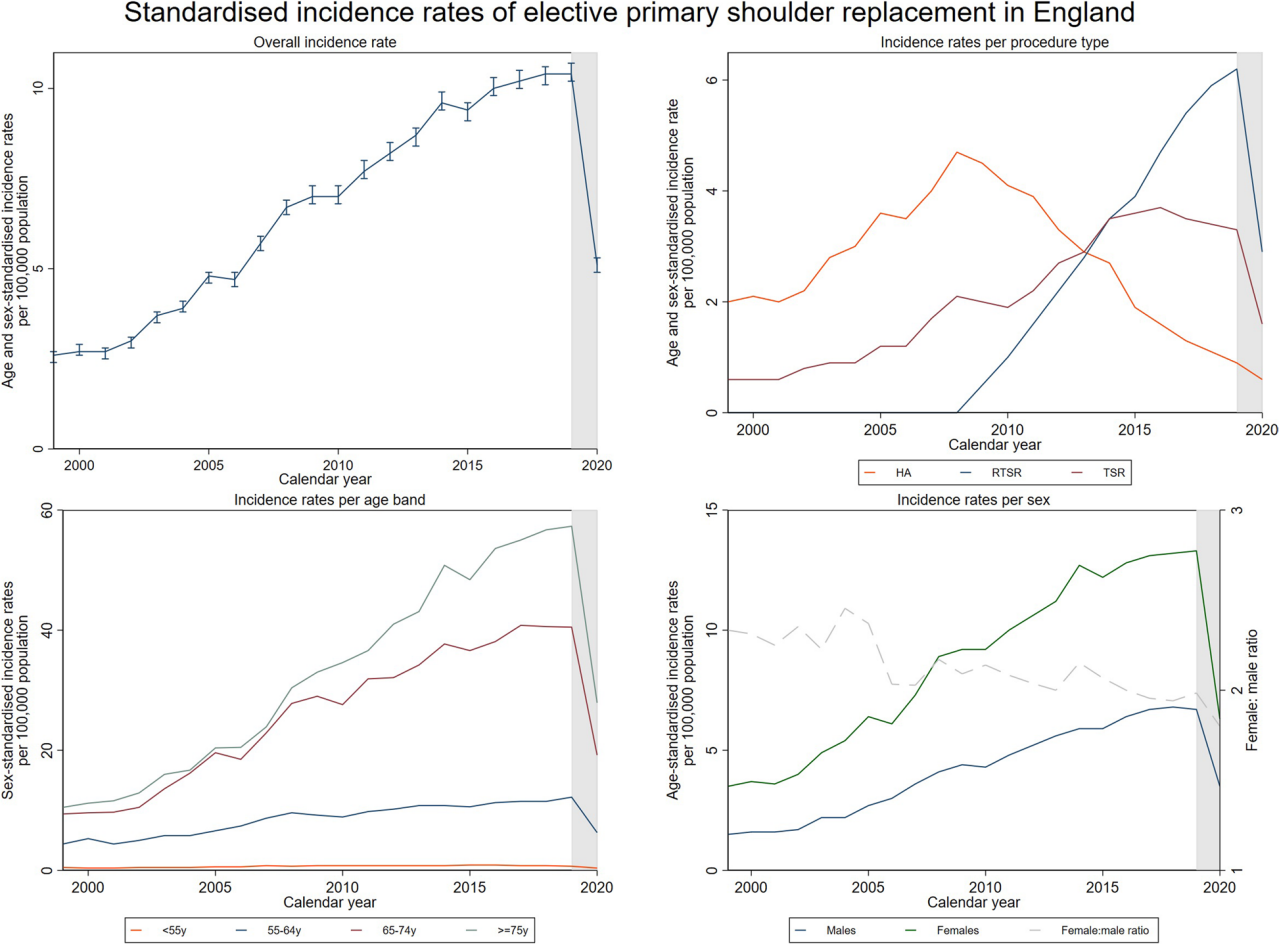
Standardised incidence rates of elective primary shoulder replacement in England. **a** Top, left: age- and sex-standardised incidence rate for each calendar year for all elective primary shoulder replacements with corresponding confidence intervals. **b** Top, right: age- and sex-standardised incidence rates for each calendar year per procedure type. RTSR, reverse total shoulder replacement; TSR, conventional total shoulder replacement; HA, hemiarthroplasty. **c** Bottom, left: sex-standardised incidence rates for each calendar year per age band. **d** Bottom, right: age-standardised incidence rates for each calendar year per sex. Shaded areas highlight the COVID-19 pandemic

Figure 1 summarises the standardised incidence rate of elective primary shoulder replacements, with an increase of 300% between 1999 and 2019 (2.6 to 10.4 per 100,000 population). During 2020, at the start of the COVID-19 pandemic, rates halved to pre-2007 levels. This increased incidence over 20 years was largely due to increased rates of surgery in patients 65 years and over, with little change in the rate of surgery in patients under 55 years. Shoulder replacements are over twice as common in females as in males, although this imbalance has slightly decreased over time. Since its adoption in 2009, usage of the RTSR rapidly increased, and in 2019 formed 60% of all shoulder replacements. The use of the HA decreased by 77% over the same 10-year period. TSR usage increased steadily until 2014 when it appeared to attain a plateau at around 3.5 procedures per 100,000 population.

### Regional trends

**Fig. 2:**
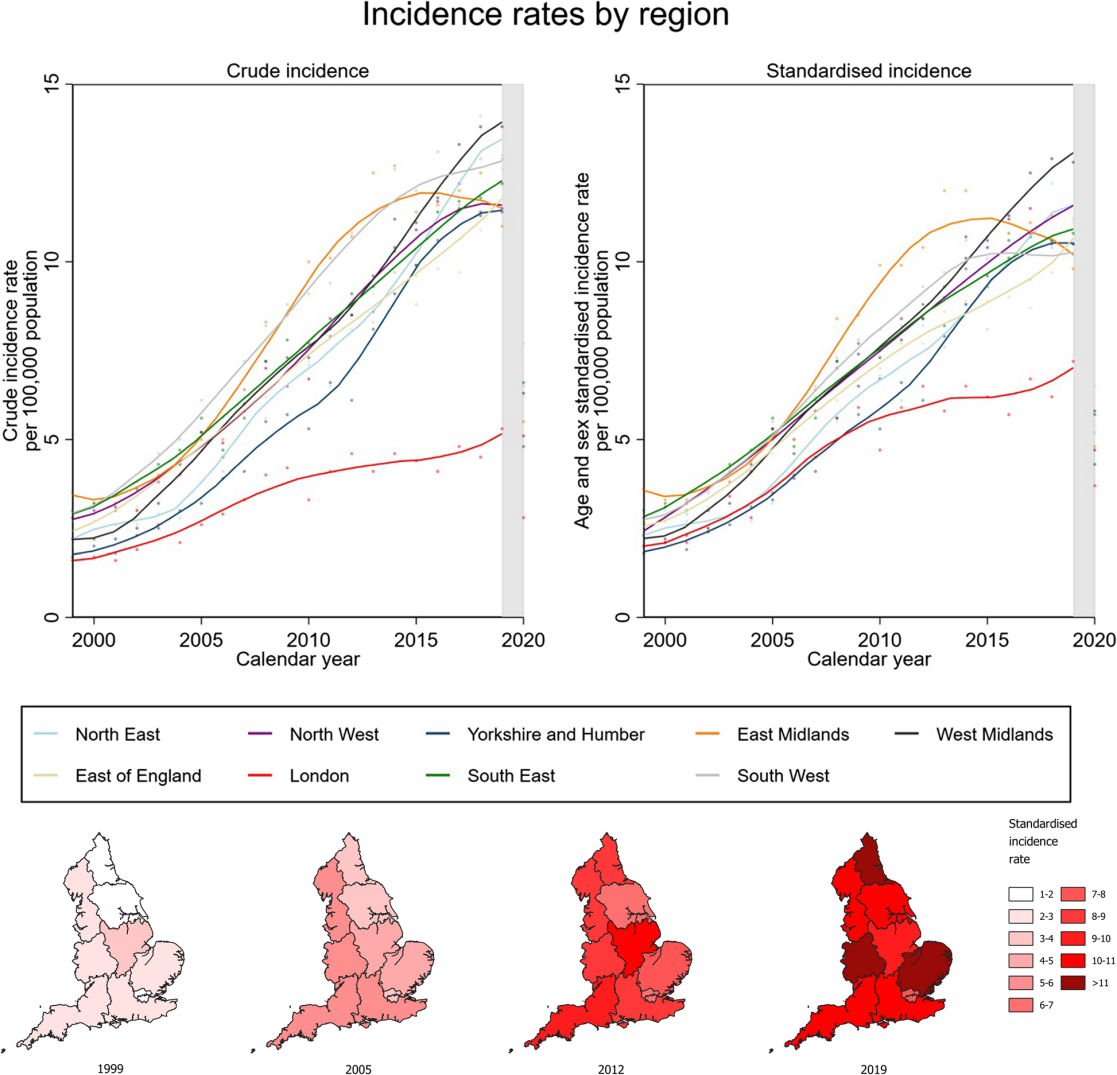
Regional variation in incidence of elective primary shoulder replacement. **a** Top, left: crude incidence rates per region. **b** Top, right: age- and sex-standardised incidence rates per region. **c** Bottom: geographical variation of age- and sex-standardised incidence rates in England. Fitted local polynomial regression lines superimposed on scatter plots to delineate trends. Shaded areas highlight the COVID-19 pandemic

Increasing incidence was observed across all regions, although the rate of increase of crude incidence was markedly lower in London (Fig. 2). However, a 38.7% reduced standardised incidence remained for the years 2013 to 2019 when compared to other regions.

**Fig. 3:**
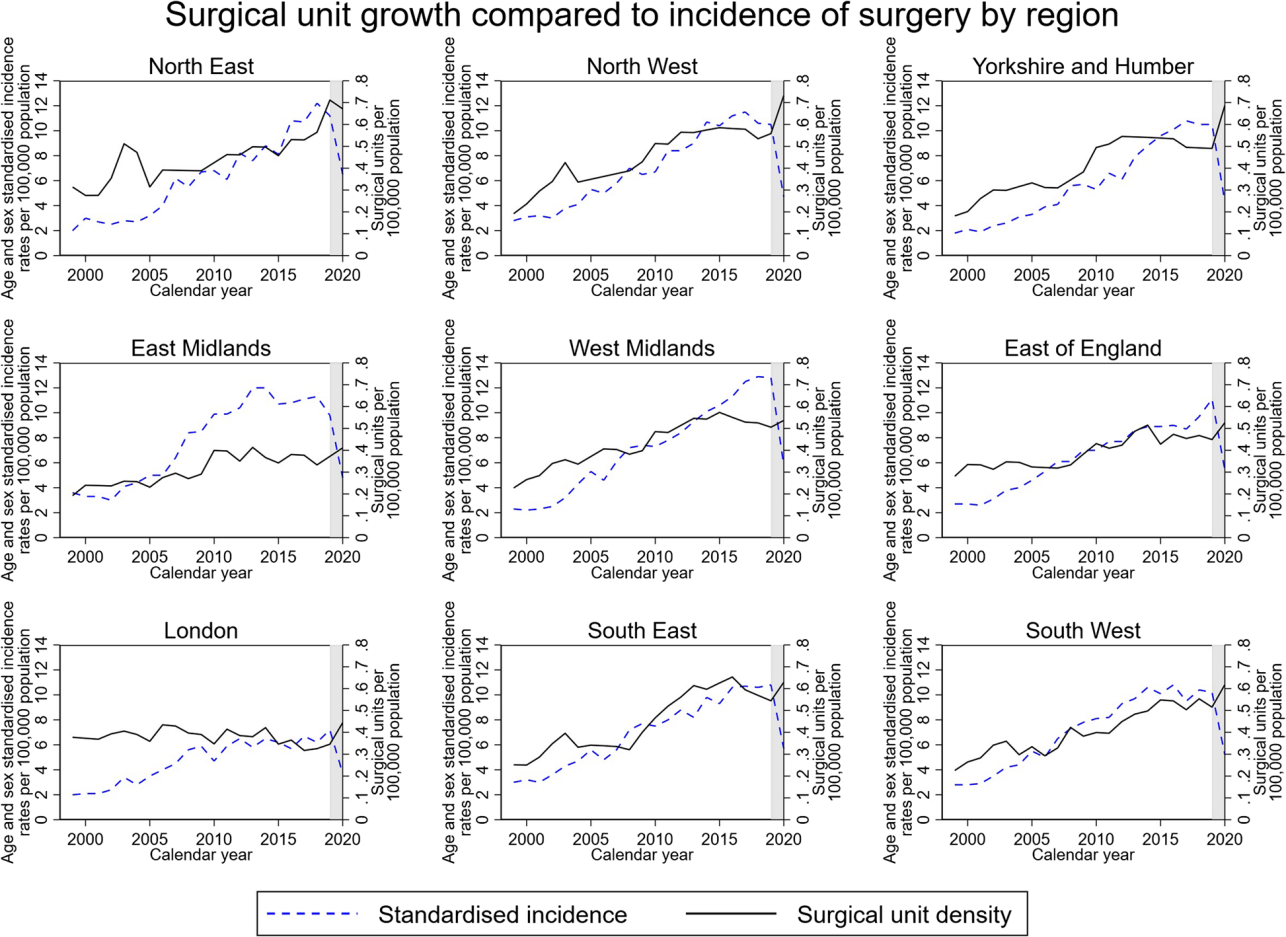
Surgical unit growth compared to incidence of surgery by region. Age- and sex-standardised incidence of elective primary procedures plotted against the surgical unit density (number of surgical units per 100,000 population) providing shoulder replacements in each region. Shaded areas highlight the COVID-19 pandemic

The relationship between the standardised incidence of elective primary shoulder replacements and the number of surgical units providing shoulder replacement surgery per population size (surgical unit density) in each region is shown in Fig. 3. In all regions apart from London, there was an increase in surgical unit density. However, the rate of growth in shoulder replacement incidence outpaced the increase in surgical unit density across all regions over time. The East Midlands appeared to have the lowest surgical unit density for its incidence of shoulder replacements. Patients residing in the East Midlands region had the greatest rate of travel to a hospital provider in a different region to get their shoulder replacement (16.8%—1 in 6), whereas over 99.5% of patients residing in the North East underwent their surgery locally (see Additional file 1).

### Socioeconomic trends

**Fig. 4:**
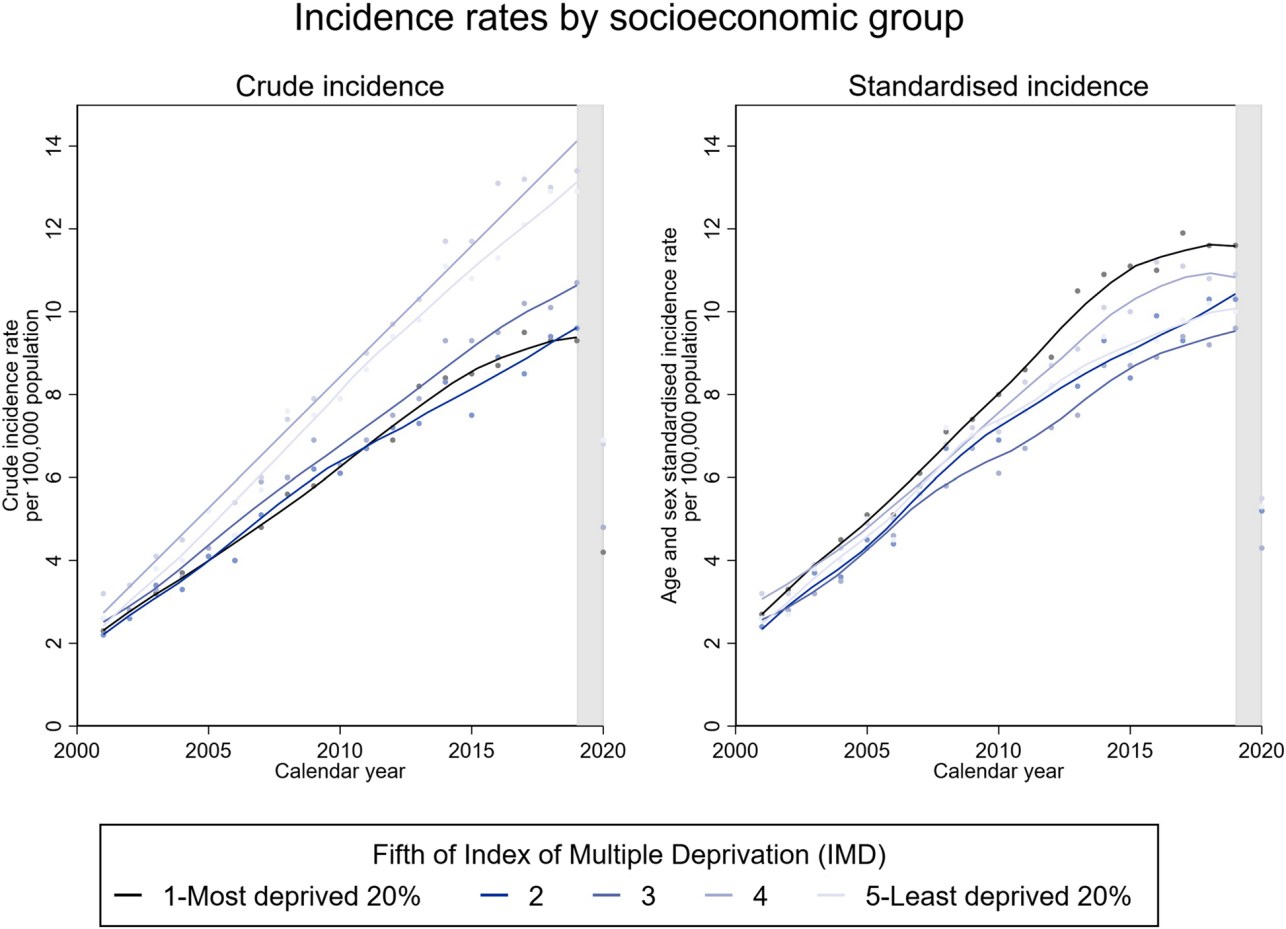
Incidence rates per socioeconomic group. **a** Left: crude incidence rates by fifth of IMD. **b** Right: age- and sex-standardised incidence rates by fifth of IMD. Fitted local polynomial regression lines superimposed on scatter plots to delineate trends. Shaded areas highlight the COVID-19 pandemic. *Note*: IMD population data only available after 2001

The crude incidence of shoulder replacements appeared greater in the least deprived 40% (IMD fifths 4 and 5), particularly in more recent years (Fig. 4). The standardised incidence increased similarly across all socioeconomic groups between 2001 and 2019.

### Patient outcomes

**Fig. 5:**
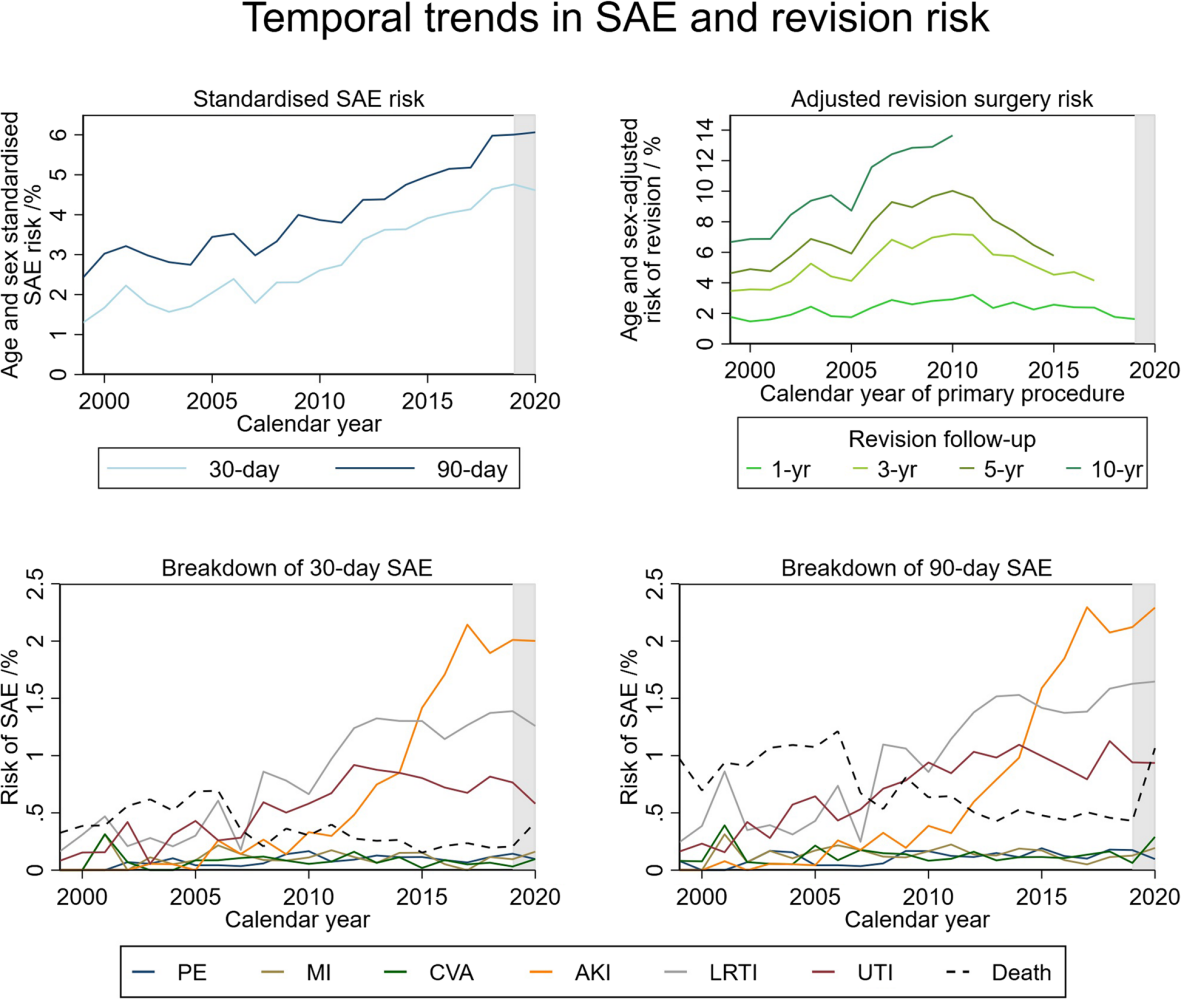
Temporal trends in SAE and revision surgery risk. **a** Top, left: age- and sex-standardised SAE risk. **b** Top, right: age- and sex-adjusted revision surgery risk. **c** Bottom, left: 30-day SAE risk by type. **d** Bottom, right: 90-day SAE risk by type. Shaded areas highlight the COVID-19 pandemic

Between 1999 and 2019, the risk of SAEs increased from 1.3 to 4.8% at 30 days and from 2.4 to 6.0% at 90 days (Fig. 5). There was little change in SAE risk during the COVID-19 pandemic in 2020. From 1999 to 2011, this increase was mainly driven by an increase in lower respiratory tract infection (LRTI) and myocardial infarction (MI). From 2012 onwards, most types of complications appeared stable apart from the risk of acute kidney injury (AKI) which increased markedly (90-day AKI risk increased by over 600% from 2011 to 2017).

The proportion of primary shoulder replacements that were revised increased between 1999 and 2010 for all follow-up periods. The greatest proportional increase during this period was for 10-year risk which more than doubled from 6.4% in 1999 to 14.1% in 2010. Thereafter, there was a reduction in revision surgery risk, although the data for longer-term follow-up was limited. There was minimal effect on the estimated risk after adjustment for age and sex (see Additional file 1).

For SAE and revision surgery risk across geographic regions, socioeconomic groups, and age bands, see Additional file 1. SAE and revision surgery risk showed a similar temporal trend across regions and socioeconomic groups. The absolute increase in SAE risk was most marked in patients aged 75 years and over, although all age bands showed an increase in SAE risk over time. Revision surgery risk was highest in younger patients under 55 years, reaching a maximum of 26.5% at 10 years follow-up in 2010, more than 2.5 times that of patients aged 75 years and over. Males had a greater risk of revision surgery at all ages. Patients in the more deprived socioeconomic groups had the highest risk of SAEs and revision including when adjusted for age and sex.

### Forecast

**Fig. 6:**
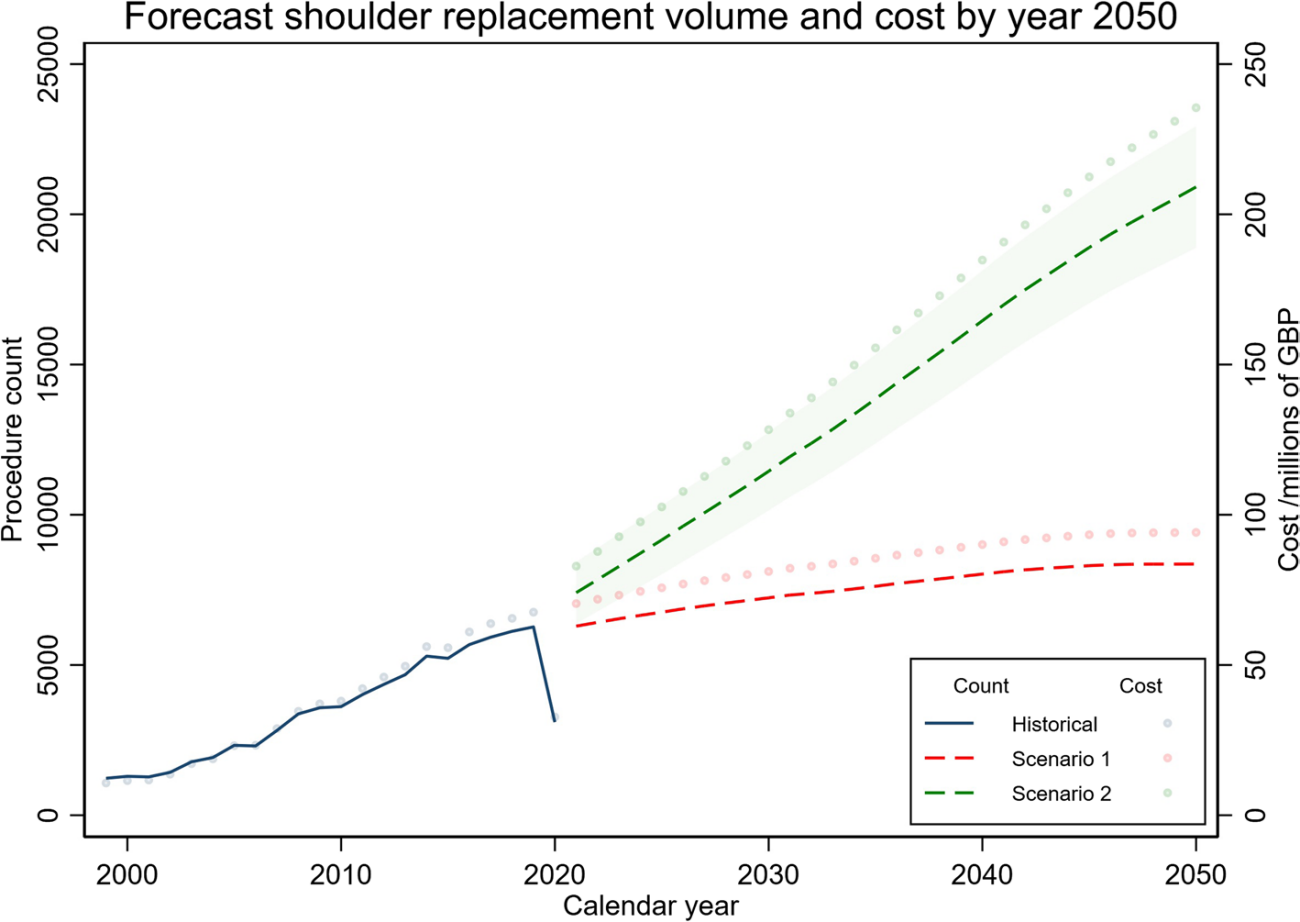
Forecast shoulder replacement volume and cost by year 2050. Lines represent the historical (solid line) and predicted (dashed lines) elective primary shoulder replacement counts in England under scenarios 1 and 2. Shaded area depicts the 95% forecast intervals for scenario 2. Dots represent historical and predicted total annual costs in 2021 GBP

The forecast burden in terms of surgical volume and expected cost following scenario 1 (incidence held constant at 2019 levels) and scenario 2 (incidence continues to increase at a linear rate based on historical trends) is shown in Fig. 6. By 2050, it is predicted that there will be 8362 and 20,912 elective primary shoulder replacements in England representing a 33% and 234% increase from 2019, under scenarios 1 and 2, respectively.

The reimbursement for an operative spell (entire hospital stay) cost the NHS an average of £11,156 (SD £1152) per shoulder replacement procedure in 2019, amounting to a total of £68 million in that year compared to £11 million in 1999. By 2050, the predicted total annual cost is £94 and £235 million for scenarios 1 and 2, respectively. Both scenarios assume a temporary, fully reversed effect of the COVID-19 pandemic on shoulder replacement volume.

## Discussion

This population-based cohort study evaluated the incidence of shoulder replacement surgery in England, reporting a 300% (4 times) increase in age- and sex-standardised incidence of elective primary shoulder replacements and a 524% (over 6 times) increase in associated hospital costs from 1999 to 2019. Between 8362 and 20,912, annual procedures are predicted by 2050, though these projections are subject to forecasting uncertainty. The standardised incidence was 38.7% lower in London between 2013 and 2019, while the East Midlands had the greatest proportion of patients (one in six) who had to travel to a different region to get their shoulder replacement. Standardising incidence for age and sex demonstrated that part of the reason for the reduced incidence in London was its younger population structure. The temporal trends in standardised incidence rates were similar across socioeconomic groups, although the average age for an elective primary shoulder replacement was 2.2 years lower in the most deprived 20% compared to the least deprived 20%. The average age at elective primary shoulder replacement increased across all regions and levels of deprivation while age at revision was more stable.

Revision surgery risk appeared to reach a maximum around 2010 after which it has steadily decreased. Patients from the more deprived socioeconomic groups had a higher risk of SAEs and revision surgery. There was a marked increase in the risk of SAEs across all regions, socioeconomic groups, age bands, and sex, reaching a maximum of 4.8% and 6.0% nationally in 2019 for 30-day and 90-day SAEs, respectively. This trend appeared to be initially driven by an increase in the risk of LRTI and MI from 1999 to 2011 followed by a considerable rise in AKI after 2011. In 2014, there was a national roll-out of a real-time electronic alert system (AKI E-Alert) to improve the detection of AKI across both primary and secondary care in the UK [24]. This likely contributed to the observed increase in AKI risk [25]. However, some studies suggest that it remains uncertain whether this rise in AKI can be entirely attributed to improved detection and that factors such as the increasing age and comorbidities of the hospital population offer another explanation [26]. Regardless of whether the rising rates of AKI are attributed to improved detection or an increase in age and comorbidities of patients, there is room for improvement in AKI prevention strategies within the population undergoing elective surgery.

There is no published study exploring long-term temporal trends of shoulder replacement surgery in the UK, and the longest study (in the USA) examined trends over 14 years [27]. An international study demonstrated the variation in incidence of shoulder replacement surgery between countries, identifying the UK as having the lowest rate among the 9 countries studied, although at the time just 2 years of registry data (2012 to 2014) was available from the UK [6]. An analysis from Germany between 2010 and 2019 reported an increase in incidence from 15 to 30 per 100,000 between 2010 and 2019, a proportional rate of increase twice that observed in our study over the same period, and expectedly double the projected rate of increase [5]. They too found a sharp increase in the proportion of RTSR procedures since 2010. Although they did not report incidence rates, another study by Villatte and colleagues considered similar projection scenarios and forecast a 31–322% increase in shoulder replacement caseloads in France over the next 30 years, similar to our predictions for England [18].

In contrast to the concerning increase in SAE risk observed in our study, Bixby and colleagues found a reduction in complication rates from 2.8 to 2.4% from 2005 to 2018 in the USA, despite noting an increase in patient comorbidity [28]. Comparing complication rates between procedures or countries is challenging, primarily due to different definitions of complications. The Scottish Arthroplasty Project recently identified an exponential rise of AKI in hip and knee replacements, reaching similar rates to ours (around 2%) in 2019, and there has been increased research activity in this area [29–31].

The strengths of this study lie in the coverage, size, and length of its national data. The universal coverage of a public healthcare system across an entire country incorporates patients from all age and socioeconomic groups, geographic regions, and levels of comorbidity. The ability to link all NHS hospital episodes for each patient ensures accurate capture of complications and revision surgery even when these occur at different hospitals in the country. Together with the size of the dataset, this coverage facilitated precise estimation of the incidence of surgery, service provision, and patient outcomes for different population subgroups. The period covered by this dataset, greater than 20 years, allowed for an assessment of long-term patient outcomes, and linked mortality data allowed for the evaluation of follow-up in patients who died before the end of the study period. The extended period of analysis also provided a more complete picture of temporal trends and improved forecast accuracy.

While HES data does capture NHS-funded work in the independent sector, it does not capture privately funded activity undertaken at independent hospitals, and data from the National Joint Registry (NJR) that does capture this activity suggests it consists of up to 10% of all elective primary shoulder replacements in the UK (LoT 2 NJR analysis team—personal communication, 2023). It follows that the true national incidence of shoulder replacements is likely up to 10% higher than that estimated by this study. Another potential limitation is that patients can request removal of their data from HES via a ‘national data opt-out’ which could result in bias or underreporting of incidence rates, although the rate at the time of dataset production was 2.6% which is likely to have little impact on the results [32]. As a hospital database, HES does not capture postoperative complications managed in the community. However, HES does capture the more serious complications, which are of greater importance to patients and the ones they expect clinicians to communicate to them. Both forecast scenarios assume that the drop in surgical volume due to the COVID-19 pandemic is temporary, and so do not take 2020 activity into consideration when predicting future rates. Indeed, there is uncertainty in the post-pandemic recovery phase of the NHS, and it may well be that any surgical backlog takes some time to clear, but the long-term projections to 2050 are unlikely to be considerably affected by this recovery phase. While we used established approaches for estimating future demand, the choice of prediction models and external factors such as policy, regulations, economic conditions, and technological advancement can always influence forecasts, meaning the forecasts estimated in this study have inherent uncertainty. Finally, while the forecasting of surgical practice is susceptible to potential major research advances in non-surgical treatment, or prevention of disease, it is unlikely that such significant medical breakthroughs will be seen in the next decade [33].

## Conclusions

Our study results carry substantial implications for healthcare providers and policymakers. The large increase in shoulder replacement incidence rates over the past two decades and the projected forecasts will impose a significant healthcare burden with associated costs set to triple (within the limitations of forecasting uncertainty) by 2050. This problem can only be addressed by adequate planning of infrastructure and workforce to accommodate the increasing demand for such orthopaedic surgery. Planners should address the regional disparities identified in this analysis. The emphasis should be on the allocation of resources to ensure the best possible care and access for every patient, irrespective of geographical location or socioeconomic background.

Despite the upsurge in shoulder replacement surgery and the accompanying growth in the healthcare experience of surgical teams and hospitals, postoperative complication rates have continued to rise. Although better diagnostics and recording of complications may partially account for this rise, current interventions have yielded limited success in decreasing these rates. There is an important need for further research and renewed efforts to understand and prevent complications after such surgery.

## Supplementary Information

**Additional file 1: Table S1.** HES OPCS operation codes for shoulder replacements. **Table S2.** HES ICD-10 codes for serious adverse events (SAE). **Table S3.** Patient region of treatment by region of residence. **Table S4.** Historic procedure counts, forecast estimates, and hospital cost. **Table S6.** Baseline characteristics for missing data. **Table S7.** Outcomes for missing data. **Fig. S1.** Data flow chart. **Fig. S2.** Average age at elective primary shoulder replacement. **Fig. S3.** Average age at revision shoulder replacement. **Fig. S4.** SAE risk by socioeconomic group. **Fig. S5.** SAE risk by region. **Fig. S6.** SAE risk by age band. **Fig. S7.** SAE risk by sex. **Fig. S8.** Breakdown of SAE risk by region. **Fig. S9.** Breakdown of SAE risk by socioeconomic group. **Fig. S10.** Crude and adjusted revision risk by region. **Fig. S11.** Crude and adjusted revision risk by socioeconomic group. **Fig. S12.** Revision rates by age band. **Fig. S13.** Revision rates by sex.

## Data Availability

The study dataset was provided by NHS Digital (DARS-NIC-432598-Q6S0C-v0.4). In accordance with NHS Digital’s Information Governance requirements; the study data cannot be shared.

## Abbreviations

AKI: Acute kidney injury
APC: Admitted Patient Care
GOR: Government Office Region
HA: Hemiarthroplasty
HES: Hospital Episode Statistics
HRG: Healthcare Resource Group
IMD: Index of Multiple Deprivation
LRTI: Lower respiratory tract infection
MI: Myocardial infarction
NHS: National Health Service
NJR: National Joint Registry
ONS: Office for National Statistics
RTSR: Reverse total shoulder replacement
SAE: Serious adverse event
TSR: Conventional total shoulder replacement
