# Supplementary material for: Shoulder replacement surgery’s rising demand, inequality of provision, and variation in outcomes: cohort study using Hospital Episode Statistics for England
Source: BMC Med. 2023 Oct 26;21:406. doi: 10.1186/s12916-023-03112-1 (PMC10601312; doi:10.1186/s12916-023-03112-1)

**Additional file 1 - Supplementary Material**

**Shoulder replacement surgery’s rising demand, inequality of provision and variation in outcomes: cohort study using Hospital Episode Statistics for England**

Contents:

[Table S1: HES OPCS operation codes for shoulder replacements 2](#_Toc169085999)

[Table S2: HES ICD-10 codes for serious adverse events (SAE) 3](#_Toc169086000)

[Table S3: Patient region of treatment by region of residence 4](#_Toc169086001)

[Table S4: Historic procedure counts, forecast estimates, and hospital cost 5](#_Toc169086002)

[Table S6: Baseline characteristics for missing data 7](#_Toc169086003)

[Figure S1: Data flow chart 8](#_Toc169086004)

[Figure S2: Average age at elective primary shoulder replacement 9](#_Toc169086005)

[Figure S3: Average age at revision shoulder replacement 10](#_Toc169086006)

[Figure S4: SAE risk by socioeconomic group 11](#_Toc169086007)

[Figure S5: SAE risk by region 12](#_Toc169086008)

[Figure S6: SAE risk by age band 13](#_Toc169086009)

[Figure S7: SAE risk by sex 14](#_Toc169086010)

[Figure S8: Breakdown of SAE risk by region 15](#_Toc169086011)

[Figure S9: Breakdown of SAE risk by socioeconomic group 16](#_Toc169086012)

[Figure S10: Crude and adjusted revision risk by region 17](#_Toc169086013)

[Figure S11: Crude and adjusted revision risk by socioeconomic group 18](#_Toc169086014)

[Figure S12: Revision rates by age band 20](#_Toc169086015)

[Figure S13: Revision rates by sex 21](#_Toc169086016)

Table S1: HES OPCS operation codes for shoulder replacements

| **Shoulder replacements** | | |  |  |
| --- | --- | --- | --- | --- |
| **Primary** | **Anatomy code** | **Type** | **Revision** | **Anatomy code** |
| W494 |  | HA | O060 |  |
| W505 |  | HA | O063 |  |
| W515 |  | HA | O073 |  |
| W581 | Z691 | HA | W430 | Z691 |
| W581 | Z813 | HA | W433 | Z685 |
| W581 | Z814 | HA | W433 | Z691 |
| W491 |  | HA | W454 | Z814 |
| W501 |  | HA | W964 |  |
| W511 |  | HA | W974 |  |
| O061 |  | TSR | W985 |  |
| O071 |  | TSR | O070 |  |
| O081 |  | TSR | W430 | Z685 |
| W431 | Z685 | TSR | W433 | Z814 |
| W431 | Z691 | TSR | W963 |  |
| W431 | Z814 | TSR | O080 |  |
| W441 | Z814 | TSR | O083 |  |
| W451 | Z814 | TSR | O084 |  |
| W961 |  | TSR | W430 | Z814 |
| W971 |  | TSR | W440 | Z814 |
| W981 |  | TSR | W443 | Z814 |
| W965 |  | RTSR | W450 | Z814 |
| W975 |  | RTSR | W453 | Z814 |
| W986 |  | RTSR | W490 |  |
|  |  |  | W493 |  |
|  |  |  | W500 |  |
|  |  |  | W503 |  |
|  |  |  | W510 |  |
|  |  |  | W513 |  |
|  |  |  | W514 |  |
|  |  |  | W580 | Z691 |
|  |  |  | W580 | Z813 |
|  |  |  | W580 | Z814 |
|  |  |  | W582 | Z691 |
|  |  |  | W582 | Z813 |
|  |  |  | W582 | Z814 |
|  |  |  | W960 |  |
|  |  |  | W966 |  |
|  |  |  | W970 |  |
|  |  |  | W973 |  |
|  |  |  | W976 |  |
|  |  |  | W980 |  |
|  |  |  | W983 |  |
|  |  |  | W984 |  |
|  |  |  | W987 |  |
|  |  |  | W054 | Z691 |
|  |  |  | W054 | Z698 |
|  |  |  | W054 | Z699 |
|  |  |  | W054 | Z813 |
|  |  |  | W054 | Z814 |
|  |  |  | W055 | Z691 |
|  |  |  | W055 | Z698 |
|  |  |  | W055 | Z699 |
|  |  |  | W055 | Z813 |
|  |  |  | W055 | Z814 |

(HA- humeral hemiarthroplasty, TSR- conventional total shoulder replacement, RTSR- reverse total shoulder replacement)

## Table S2: HES ICD-10 codes for serious adverse events (SAE)

| **Event** | **ICD-10 codes** |
| --- | --- |
| Pulmonary embolism | I26 |
| Myocardial infarction | I21,I22 |
| Cerebrovascular event | I60,I61,I62,I63,I64 |
| Acute kidney injury | N17 |
| Lower respiratory tract infection | J12,J13,J14,J15,J16,J18,J22,J86,J440,J851,J690 |
| Urinary tract infection | N10,N300,N308,N309,N390 |
| Death | *Civil Registration Mortality data linked to HES data* |

## Table S3: Patient region of treatment by region of residence

| **Treatment region**^§^ | **Region of residence** | | | | | | | | |
| --- | --- | --- | --- | --- | --- | --- | --- | --- | --- |
|  | North East | North West | Yorkshire and Humber | East Midlands | West Midlands | East of England | London | South East | South West |
| North East (n) | 3,847 | * | * | * | * | * | * | * | * |
| (%) | **99.53** | * | * | * | * | * | * | * | * |
| North West (n) | * | 10,487 | * | * | * | * | * | * | * |
| (%) | * | **97.19** | * | * | * | * | * | * | * |
| Yorkshire and Humber (n) | * | * | 6,389 | * | * | * | * | * | * |
| (%) | * | * | **93.16** | * | * | * | * | * | * |
| East Midlands (n) | * | * | * | 6,591 | * | * | * | * | * |
| (%) | * | * | * | **83.23** | * | * | * | * | * |
| West Midlands (n) | * | * | * | * | 8,604 | * | * | * | * |
| (%) | * | * | * | * | **95.52** | * | * | * | * |
| East of England (n) | * | * | * | * | * | 7,927 | * | * | * |
| (%) | * | * | * | * | * | **90.19** | * | * | * |
| London (n) | * | * | * | * | * | * | 5,874 | * | * |
| (%) | * | * | * | * | * | * | **95.87** | * | * |
| South East (n) | * | * | * | * | * | * | * | 12,657 | * |
| (%) | * | * | * | * | * | * | * | **91.72** | * |
| South West (n) | * | * | * | * | * | * | * | * | 9,378 |
| (%) | * | * | * | * | * | * | * | * | **97.74** |

§ Missing treatment region in 342 (0.4%) of patients so complete case data shown.

* Only percentages for same region of residence and treatment shown in order to prevent the disclosure of small numbers at a sub-national level. The percentage is calculated based on the proportion of patients who both reside and receive treatment within the same region, with the numerator reflecting the number of such patients and the denominator representing the total number of patients living in that region.

## Table S4: Historic procedure counts, forecast estimates, and hospital cost

| Year | Cost- historic /million GBP | Cost- Scenario 1 /million GBP | Cost- Scenario 2 /million GBP | Count- historic | Count- Scenario 1 | Count- Scenario 2 |
| --- | --- | --- | --- | --- | --- | --- |
| 1999 | 10.7 |  |  | 1231 |  |  |
| 2000 | 11.5 |  |  | 1295 |  |  |
| 2001 | 11.7 |  |  | 1277 |  |  |
| 2002 | 13.7 |  |  | 1428 |  |  |
| 2003 | 17.2 |  |  | 1779 |  |  |
| 2004 | 18.8 |  |  | 1921 |  |  |
| 2005 | 23.1 |  |  | 2326 |  |  |
| 2006 | 23.3 |  |  | 2307 |  |  |
| 2007 | 28.8 |  |  | 2823 |  |  |
| 2008 | 34.6 |  |  | 3373 |  |  |
| 2009 | 37.1 |  |  | 3578 |  |  |
| 2010 | 38.0 |  |  | 3615 |  |  |
| 2011 | 42.2 |  |  | 4016 |  |  |
| 2012 | 46.0 |  |  | 4355 |  |  |
| 2013 | 49.6 |  |  | 4679 |  |  |
| 2014 | 56.1 |  |  | 5296 |  |  |
| 2015 | 55.8 |  |  | 5222 |  |  |
| 2016 | 61.0 |  |  | 5680 |  |  |
| 2017 | 63.8 |  |  | 5925 |  |  |
| 2018 | 65.5 |  |  | 6121 |  |  |
| 2019 | 67.6 |  |  | 6268 |  |  |
| 2020 | 32.7 |  |  | 3098 |  |  |
| 2021 |  | 70.5 | 82.9 |  | 6294 | 7408 |
| 2022 |  | 71.9 | 87.8 |  | 6420 | 7842 |
| 2023 |  | 73.2 | 92.7 |  | 6541 | 8281 |
| 2024 |  | 74.5 | 97.7 |  | 6652 | 8720 |
| 2025 |  | 75.7 | 102.6 |  | 6759 | 9162 |
| 2026 |  | 77.0 | 107.8 |  | 6870 | 9622 |
| 2027 |  | 78.1 | 112.8 |  | 6969 | 10068 |
| 2028 |  | 79.1 | 117.9 |  | 7060 | 10518 |
| 2029 |  | 80.1 | 123.0 |  | 7147 | 10976 |
| 2030 |  | 81.1 | 128.3 |  | 7237 | 11448 |
| 2031 |  | 82.2 | 133.9 |  | 7330 | 11937 |
| 2032 |  | 82.9 | 138.9 |  | 7385 | 12381 |
| 2033 |  | 83.6 | 144.2 |  | 7454 | 12849 |
| 2034 |  | 84.6 | 149.8 |  | 7535 | 13345 |
| 2035 |  | 85.5 | 155.6 |  | 7620 | 13857 |
| 2036 |  | 86.6 | 161.5 |  | 7713 | 14389 |
| 2037 |  | 87.4 | 167.2 |  | 7788 | 14893 |
| 2038 |  | 88.3 | 172.9 |  | 7865 | 15405 |
| 2039 |  | 89.2 | 178.8 |  | 7946 | 15929 |
| 2040 |  | 90.1 | 184.8 |  | 8027 | 16457 |
| 2041 |  | 91.0 | 190.8 |  | 8105 | 16987 |
| 2042 |  | 91.7 | 196.5 |  | 8169 | 17491 |
| 2043 |  | 92.3 | 201.9 |  | 8217 | 17963 |
| 2044 |  | 92.9 | 207.2 |  | 8264 | 18433 |
| 2045 |  | 93.4 | 212.5 |  | 8306 | 18896 |
| 2046 |  | 93.8 | 217.5 |  | 8338 | 19339 |
| 2047 |  | 94.0 | 222.2 |  | 8353 | 19744 |
| 2048 |  | 94.0 | 226.6 |  | 8359 | 20133 |
| 2049 |  | 94.1 | 231.0 |  | 8360 | 20517 |
| 2050 |  | 94.1 | 235.5 |  | 8362 | 20912 |

## Table S6: Baseline characteristics for missing data

| **Characteristic** | **Non-missing** | **Missing** |
| --- | --- | --- |
|  | **(n=77,613)** | **(n=1,029)** |
| Age, mean (SD),y | 71.1 (10.7) | 68.9 (12.0) |
| Sex |  |  |
| Male | 22,739 (29.3) | 313 (30.4) |
| Female | 54,874 (70.7) | 716 (69.6) |
| Year of primary |  |  |
| 1999 | 1,231 (96.6) | 44 (3.5) |
| 2000 | 1,295 (97.8) | 29 (2.2) |
| 2001 | 1,277 (98.0) | 26 (2.0) |
| 2002 | 1,428 (99.1) | 13 (0.9) |
| 2003 | 1,779 (98.7) | 24 (1.3) |
| 2004 | 1,921 (99.1) | 17 (0.9) |
| 2005 | 2,326 (98.7) | 30 (1.3) |
| 2006 | 2,307 (98.6) | 32 (1.4) |
| 2007 | 2,823 (98.7) | 38 (1.3) |
| 2008 | 3,373 (98.8) | 42 (1.2) |
| 2009 | 3,578 (98.7) | 47 (1.3) |
| 2010 | 3,615 (98.5) | 54 (1.5) |
| 2011 | 4,016 (98.7) | 55 (1.4) |
| 2012 | 4,355 (98.8) | 53 (1.2) |
| 2013 | 4,679 (98.7) | 59 (1.3) |
| 2014 | 5,296 (98.9) | 61 (1.1) |
| 2015 | 5,222 (98.5) | 82 (1.6) |
| 2016 | 5,680 (98.6) | 82 (1.4) |
| 2017 | 5,925 (98.8) | 72 (1.2) |
| 2018 | 6,121 (99.0) | 63 (1.0) |
| 2019 | 6,268 (98.8) | 78 (1.2) |
| 2020 | 3,098 (99.1) | 28 (0.9) |

Percentages given in brackets for year of primary represents proportions of the total number of procedures that year. Percentages given in brackets for sex represent proportions of the total number of cases in the cohort.

## Figure S1: Data flow chart


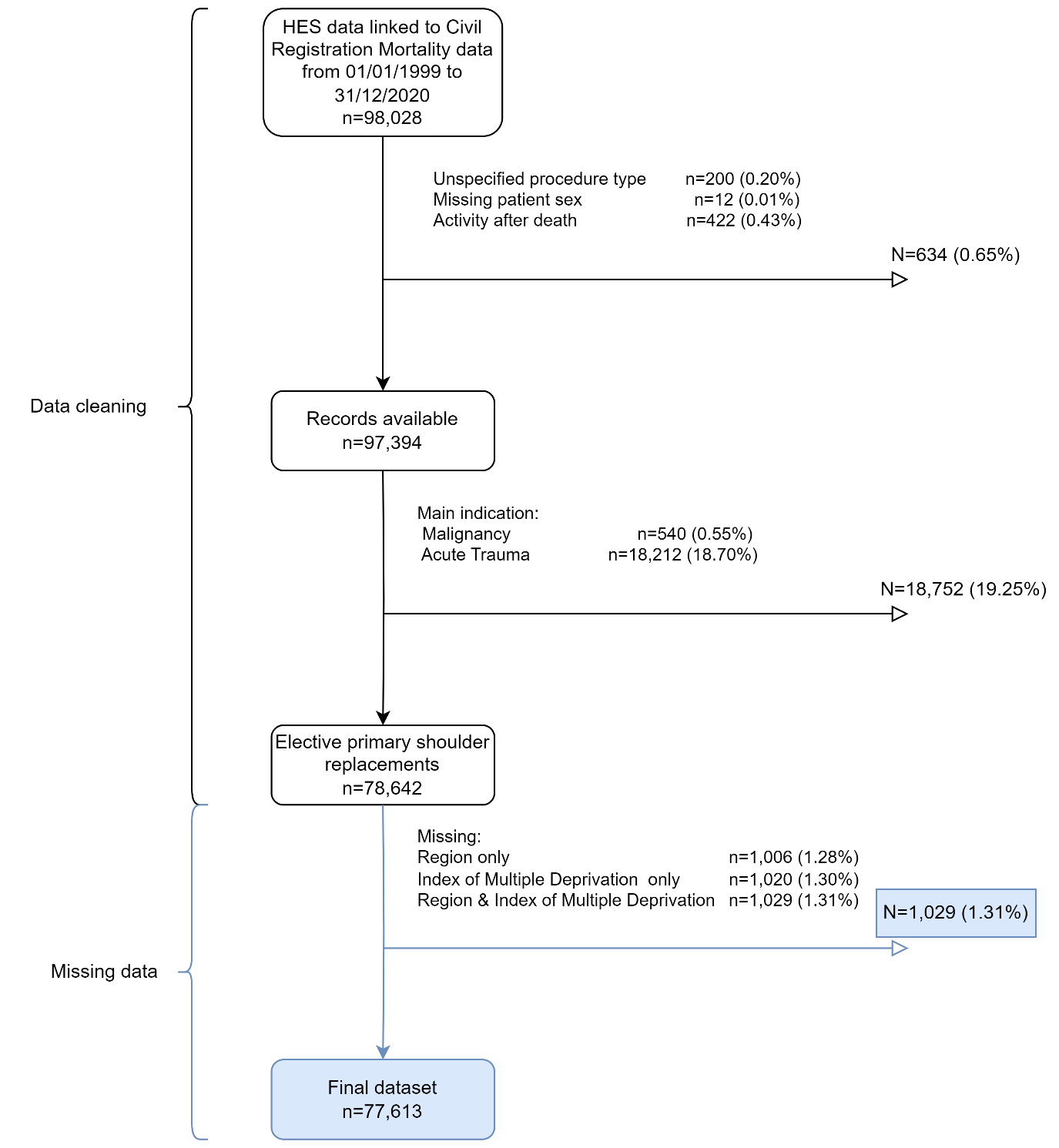


## Figure S2: Average age at elective primary shoulder replacement


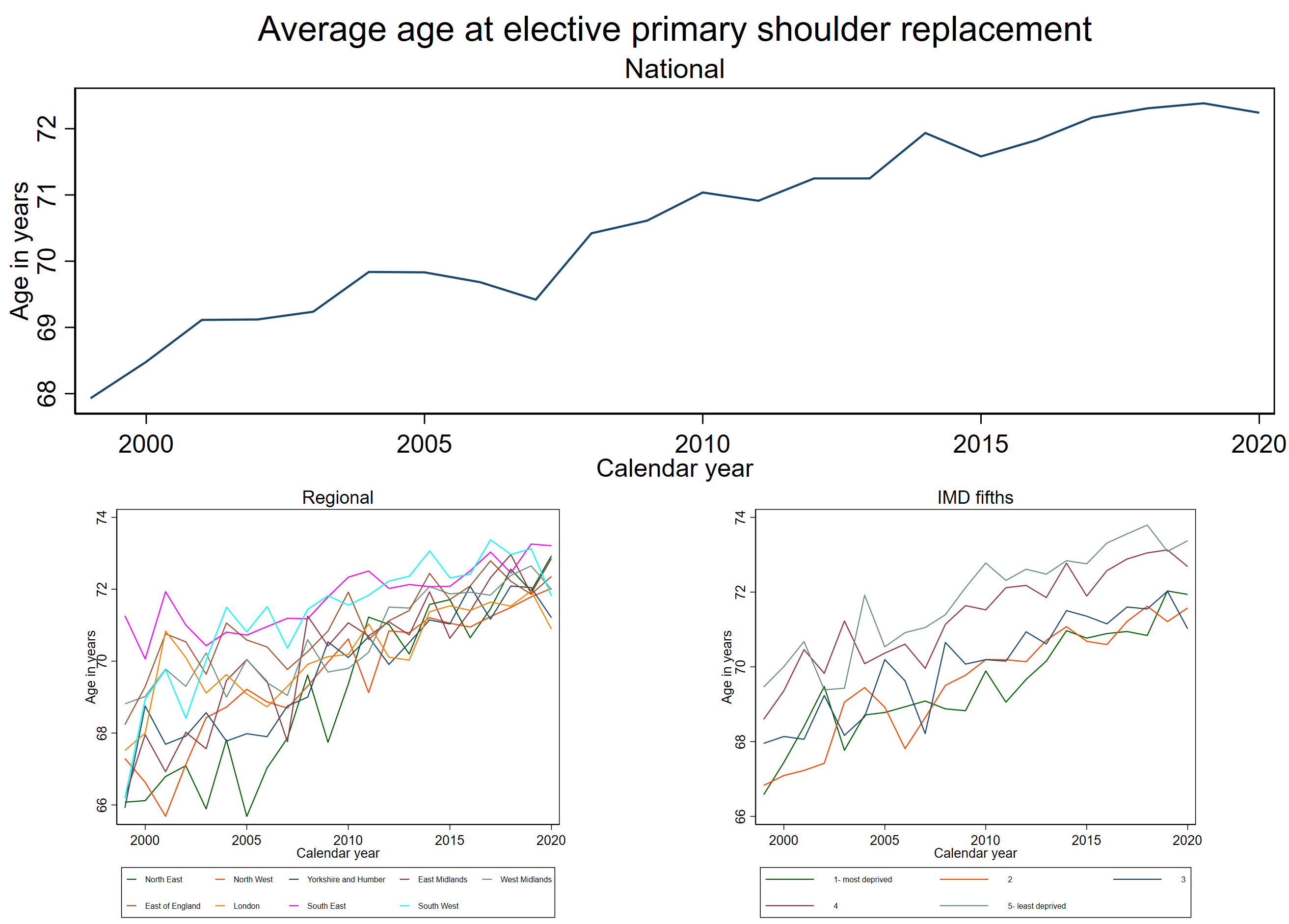


## Figure S3: Average age at revision shoulder replacement


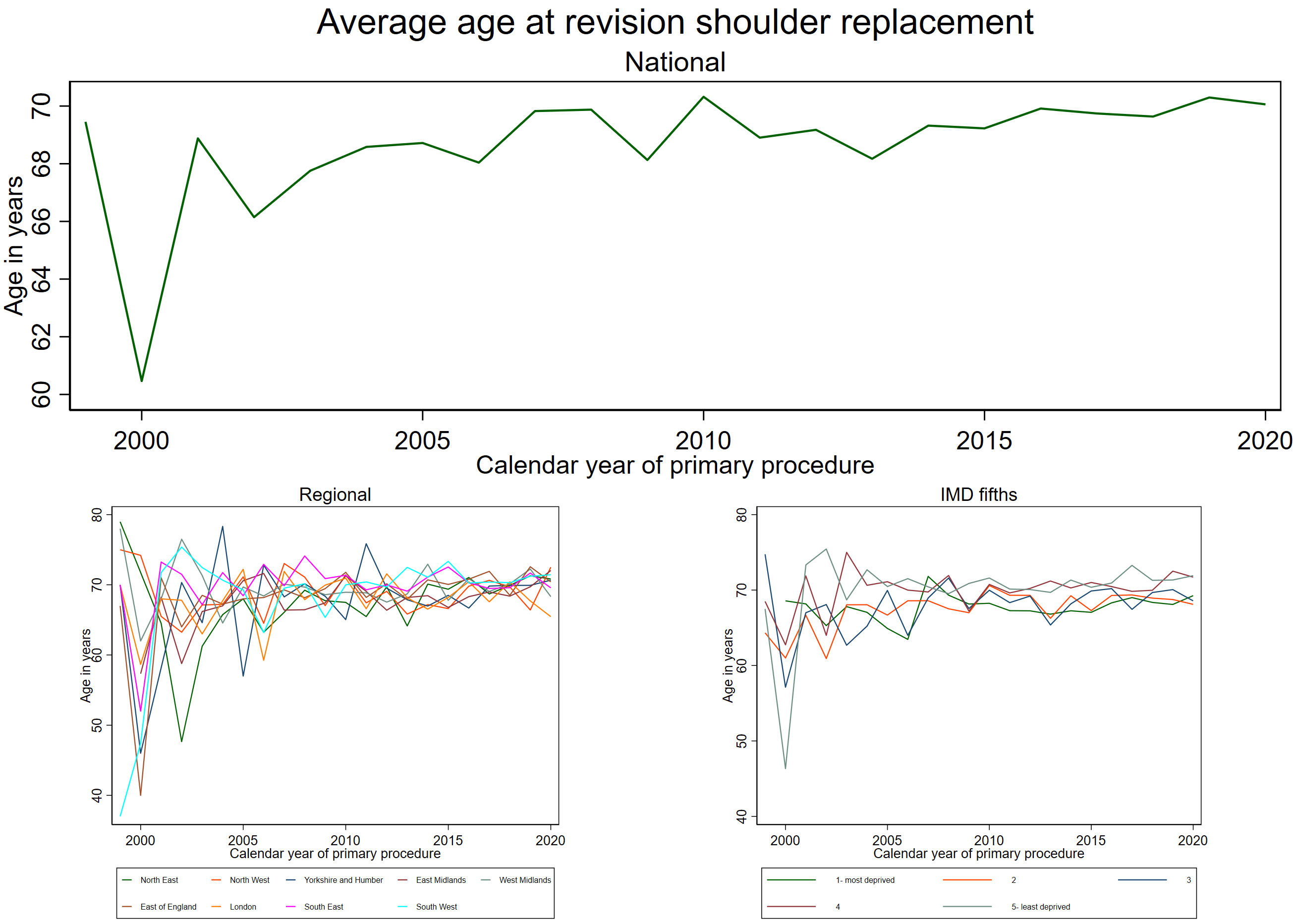


## Figure S4: SAE risk by socioeconomic group


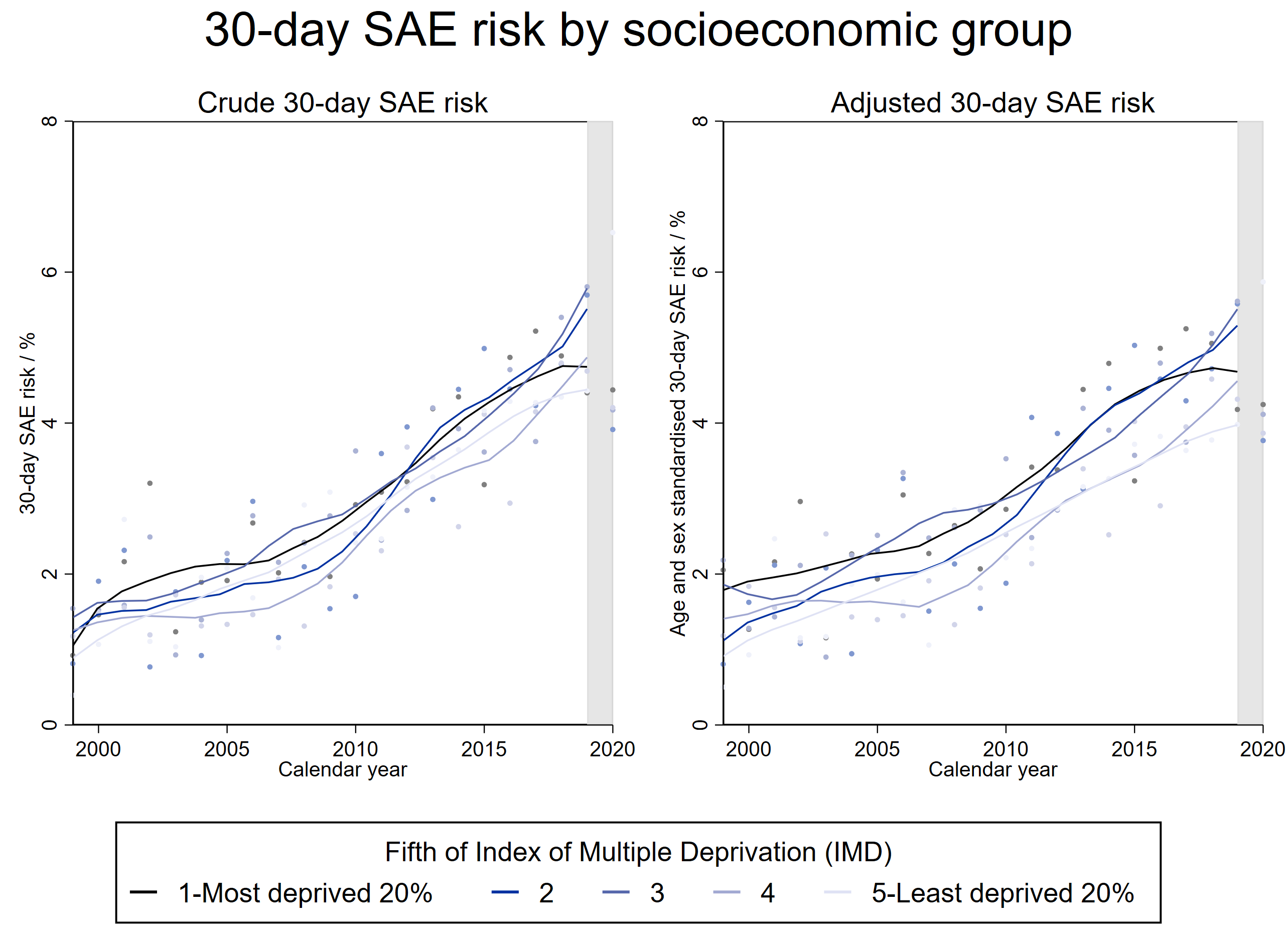


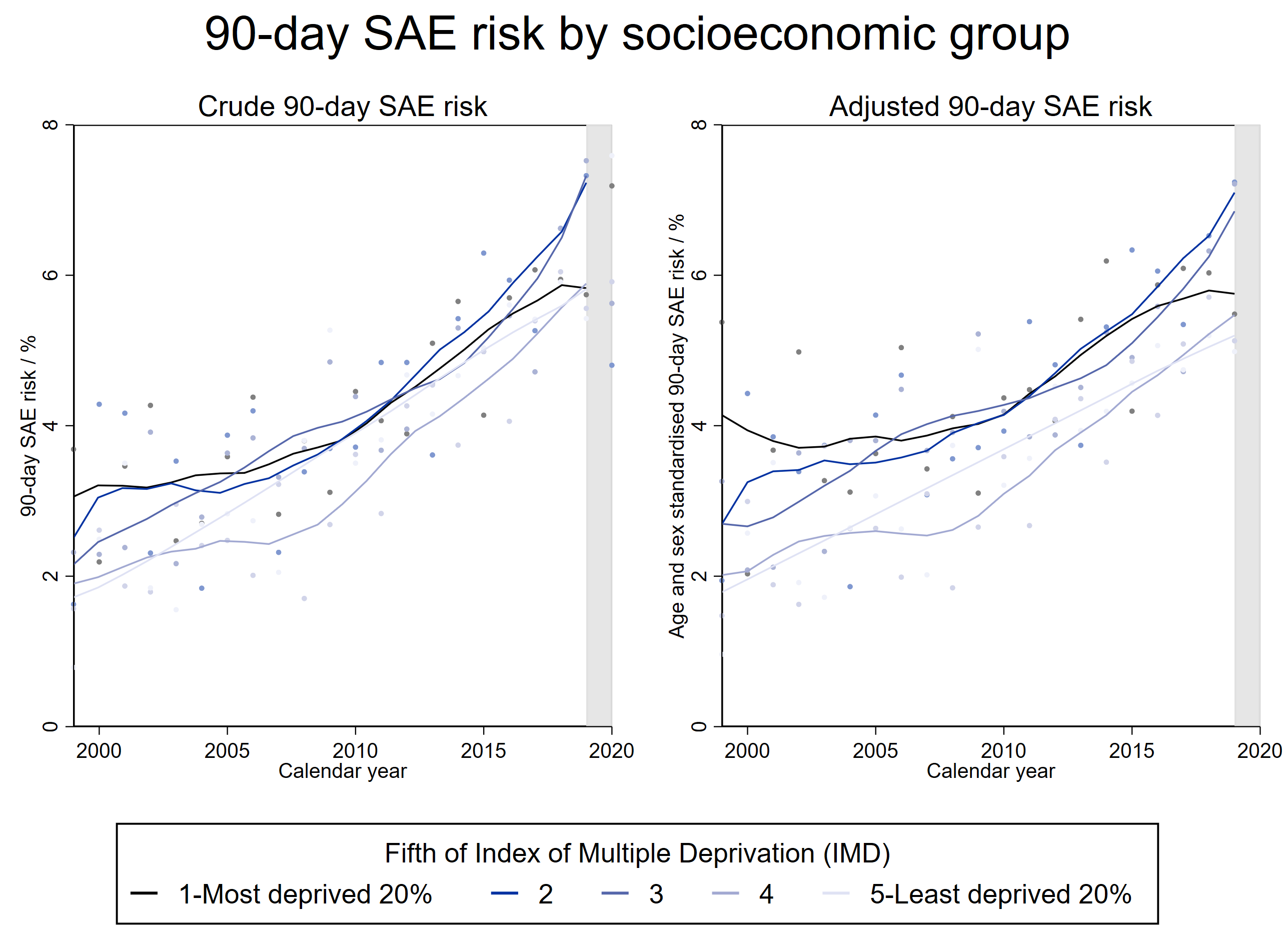


## Figure S5: SAE risk by region


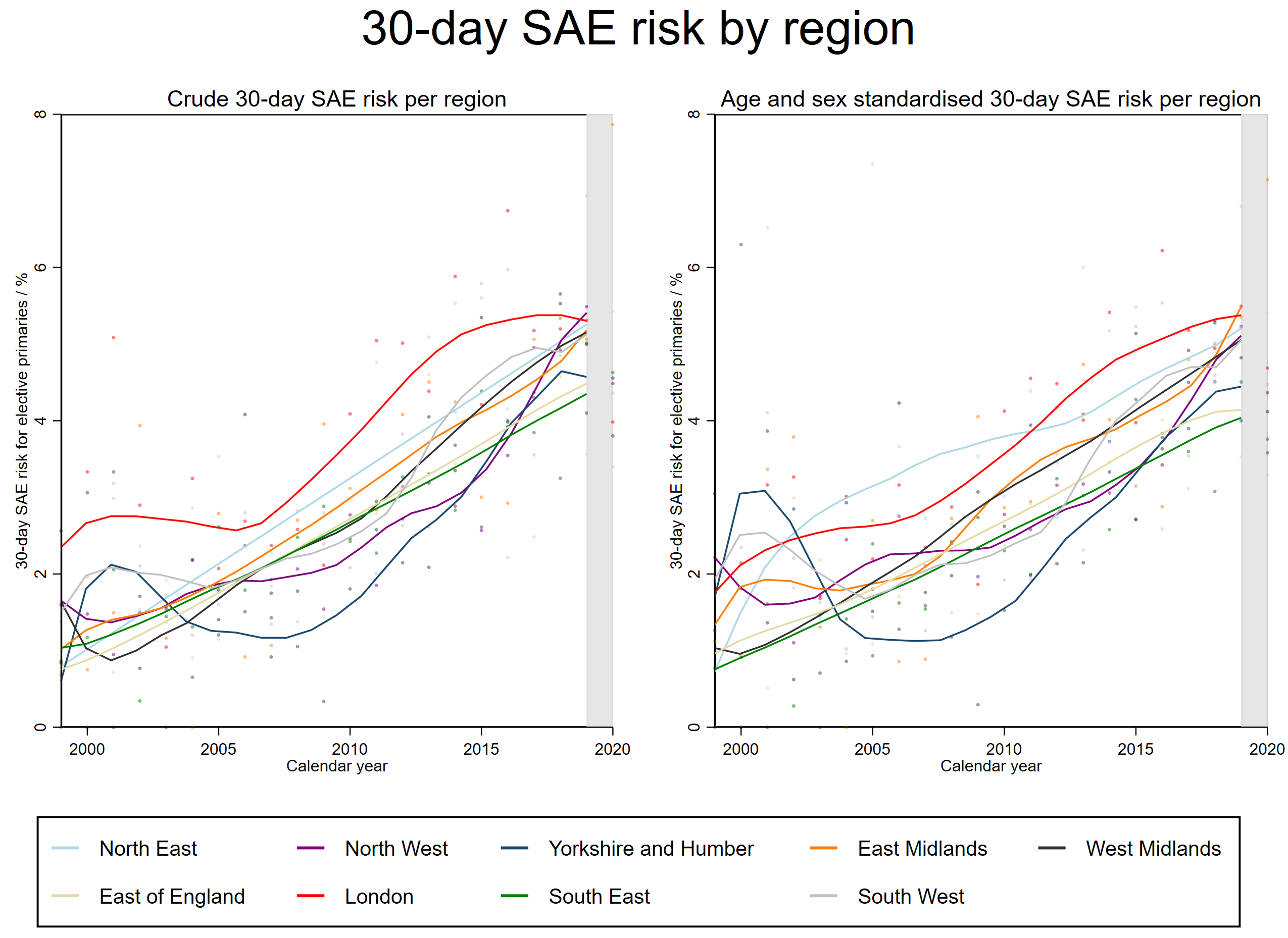


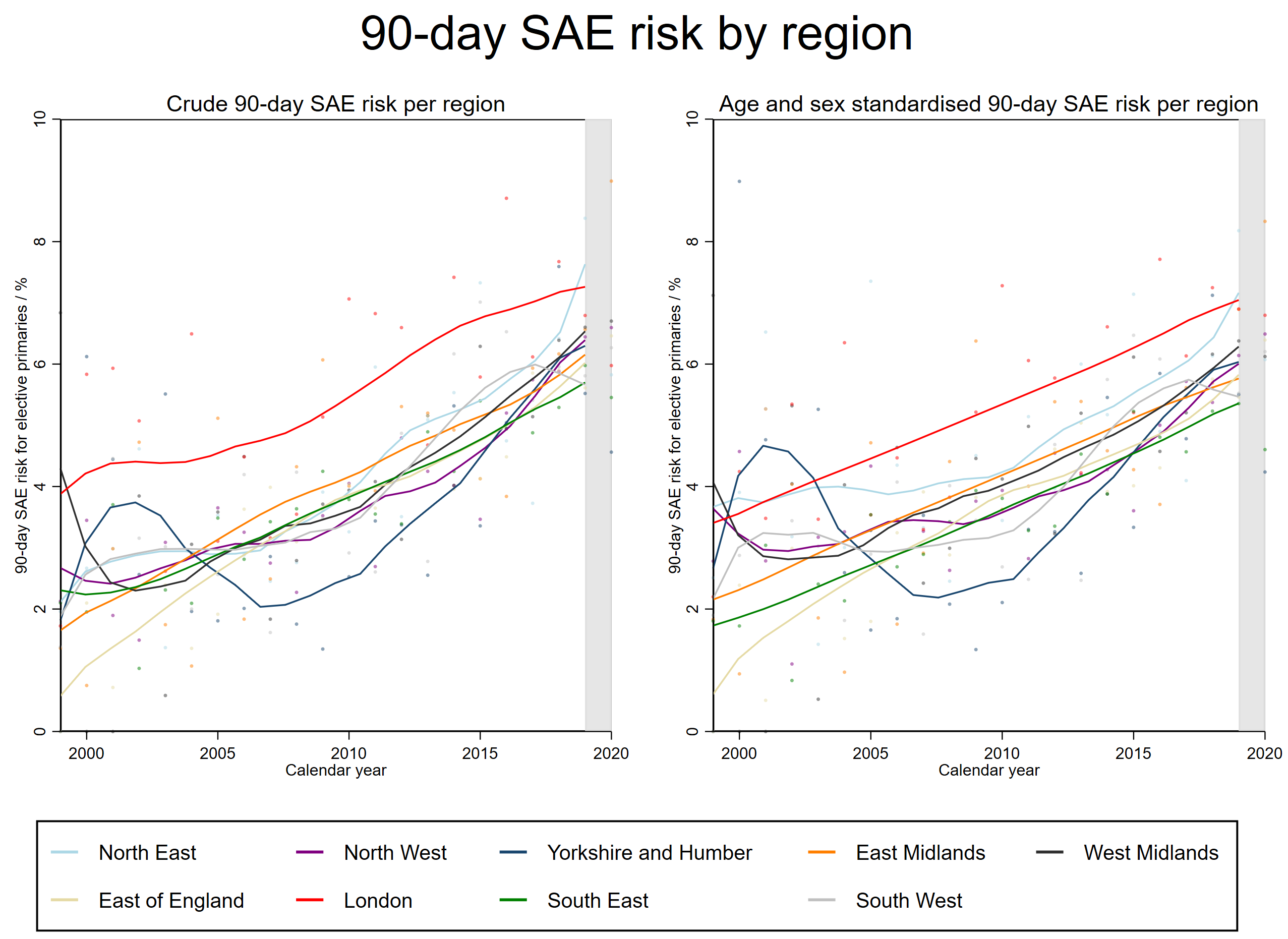


## Figure S6: SAE risk by age band


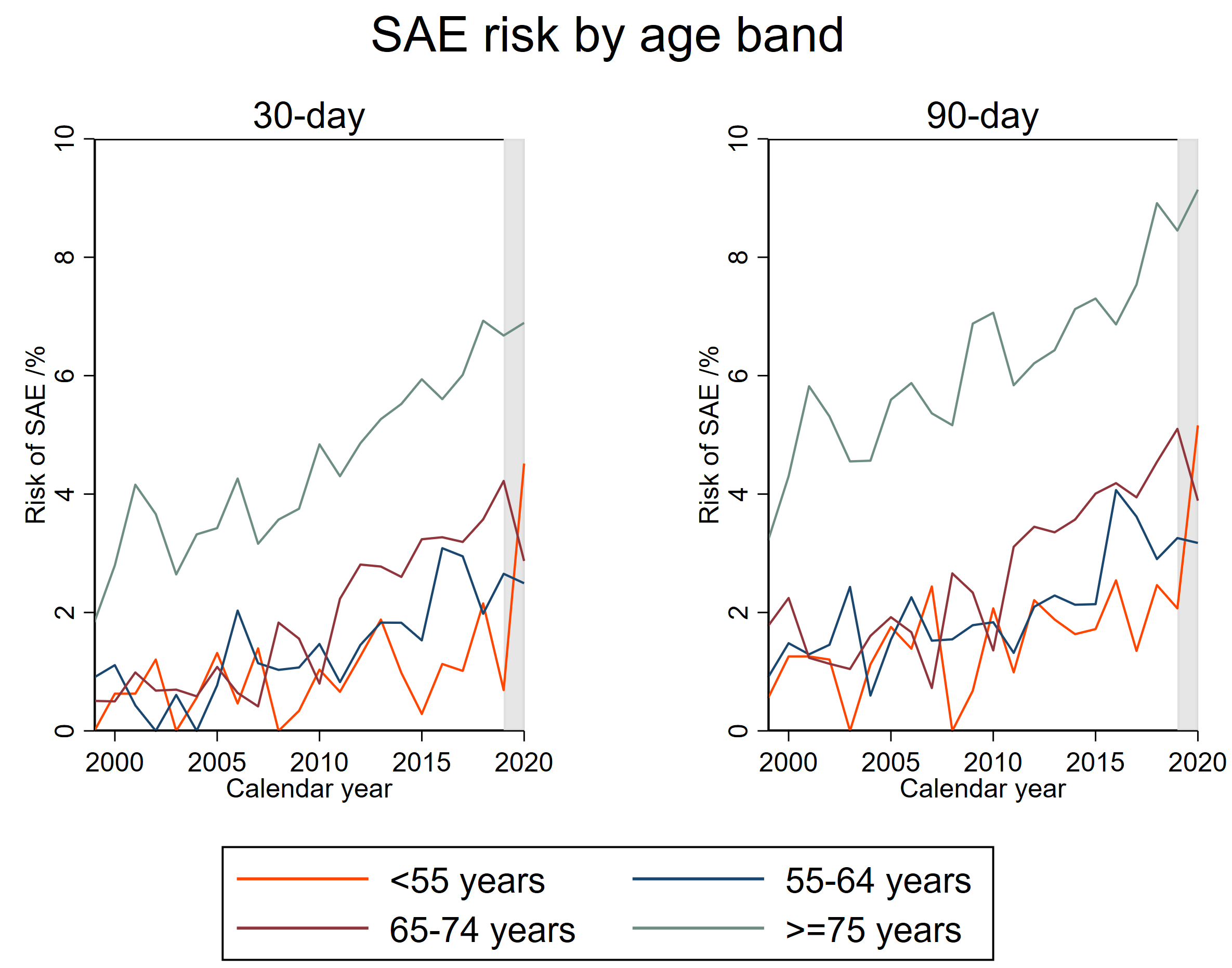


## Figure S7: SAE risk by sex


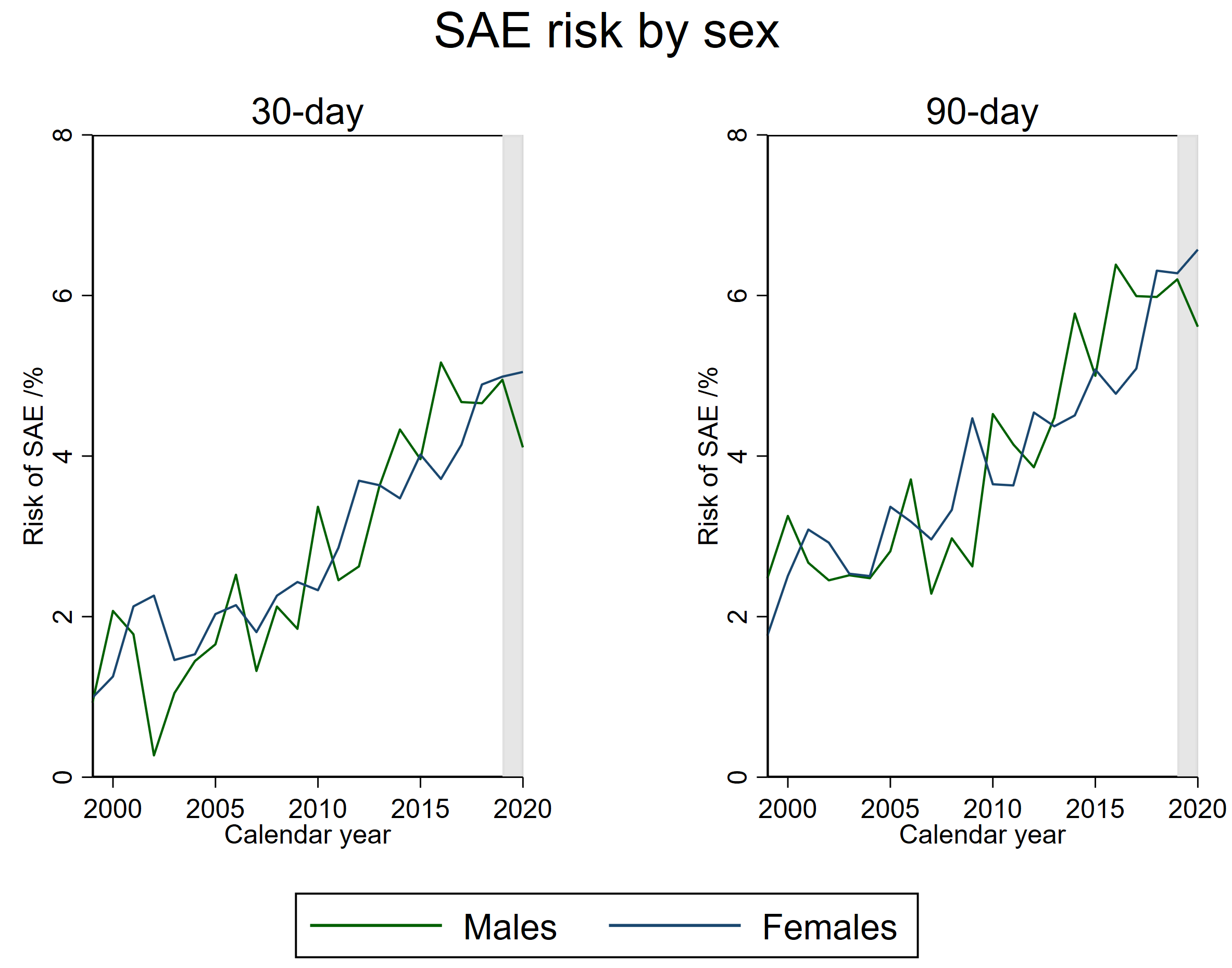


## Figure S8: Breakdown of SAE risk by region

**
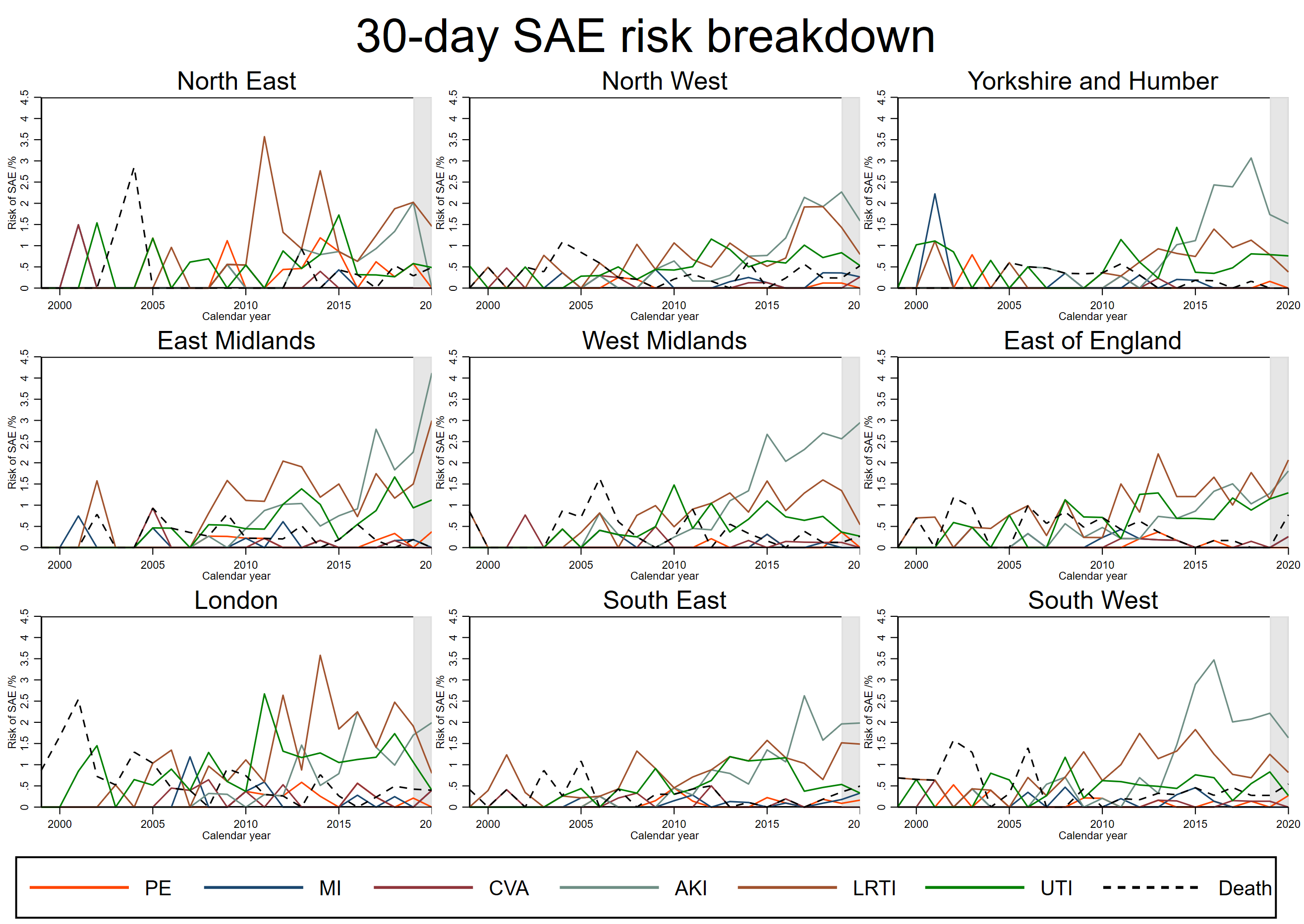
**

**
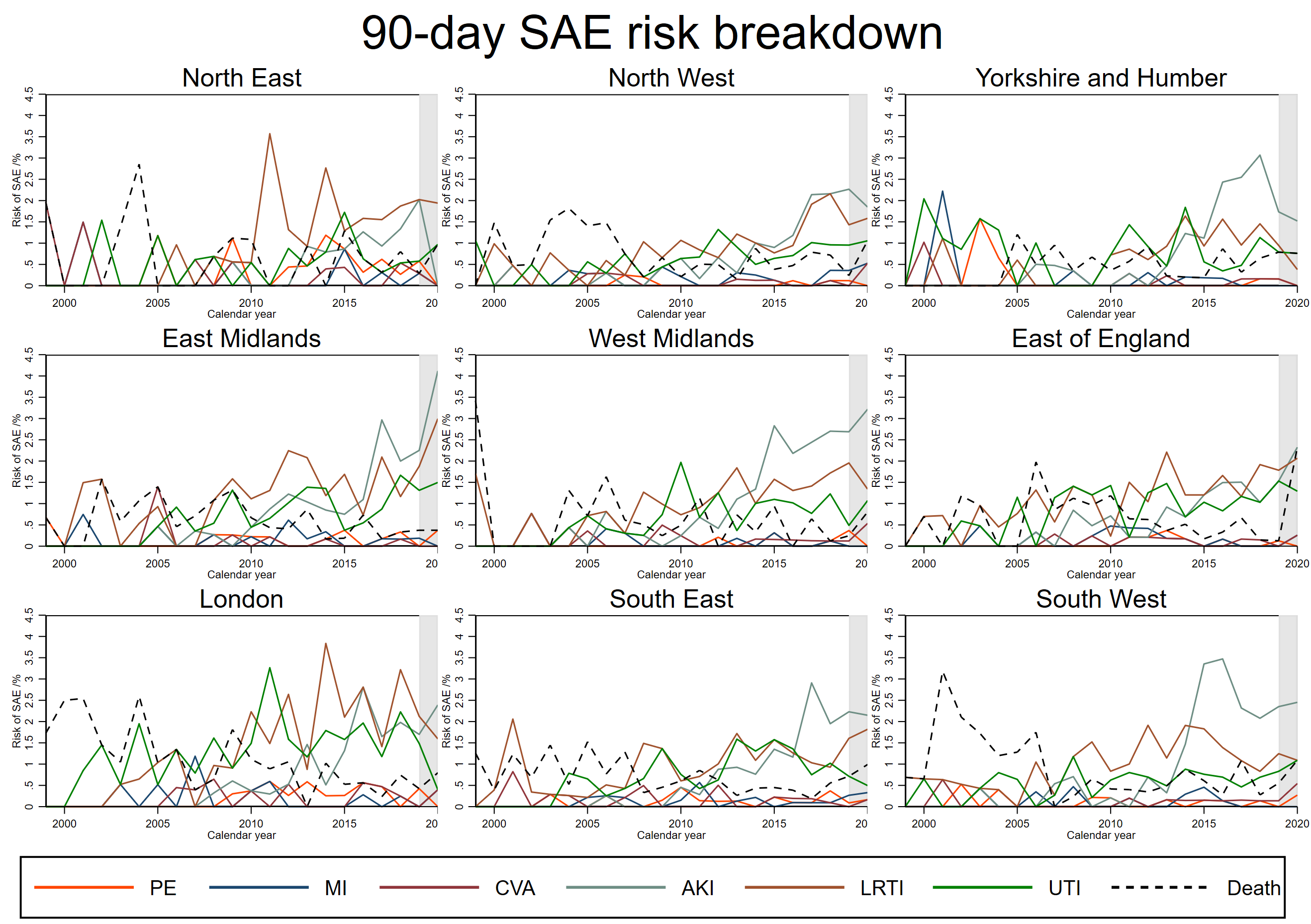
**

## Figure S9: Breakdown of SAE risk by socioeconomic group

**
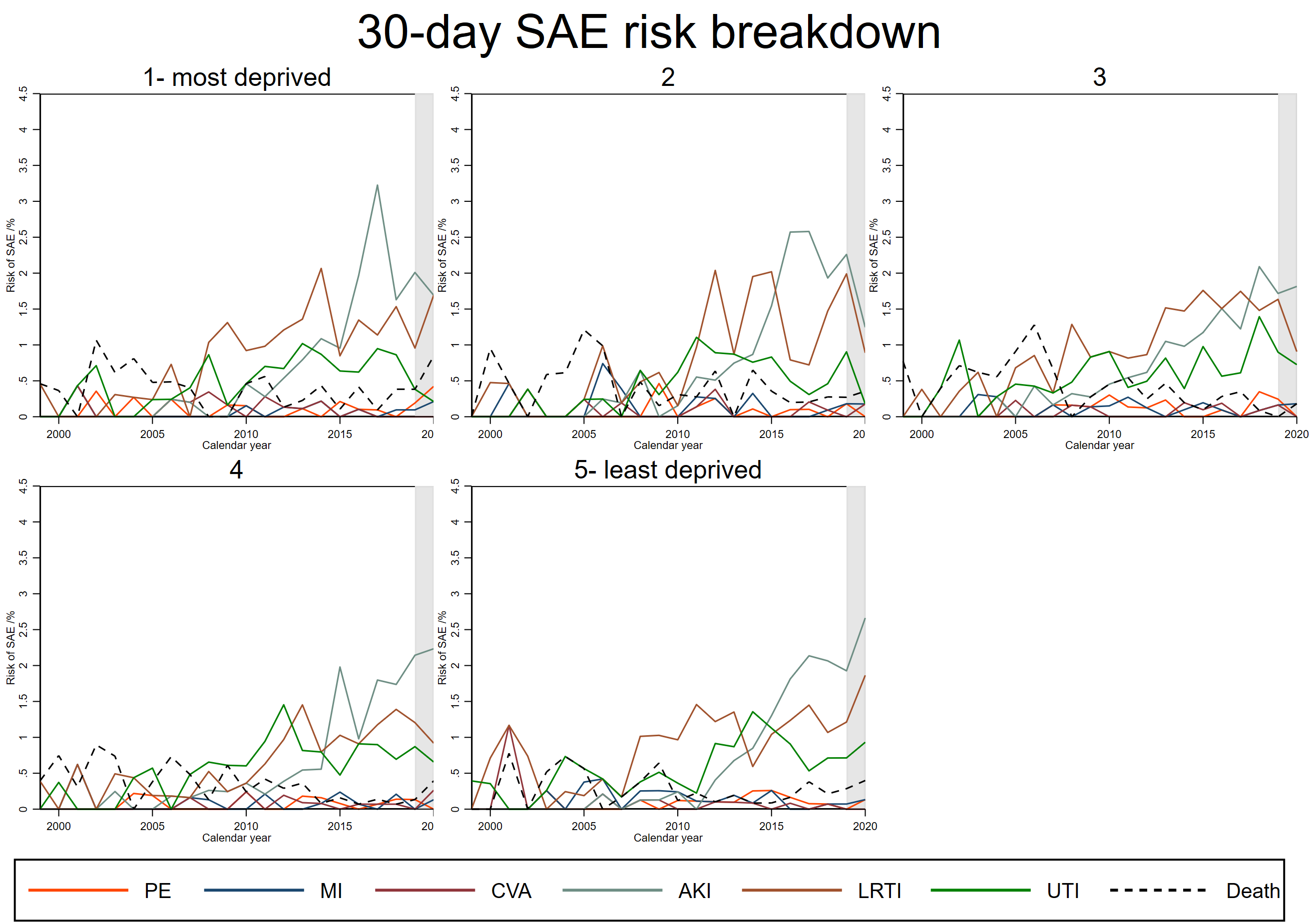
**

**
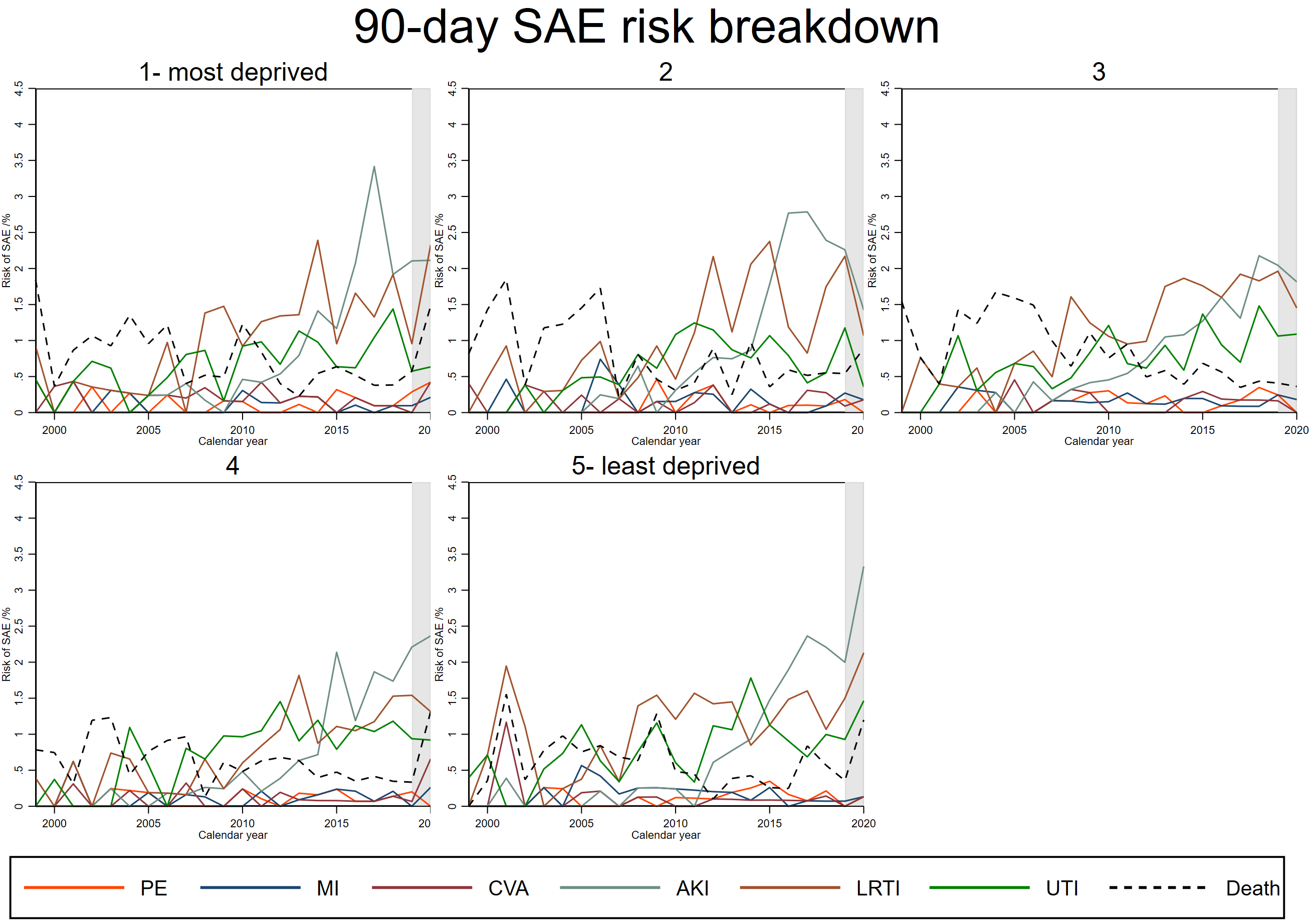
**

## Figure S10: Crude and adjusted revision risk by region


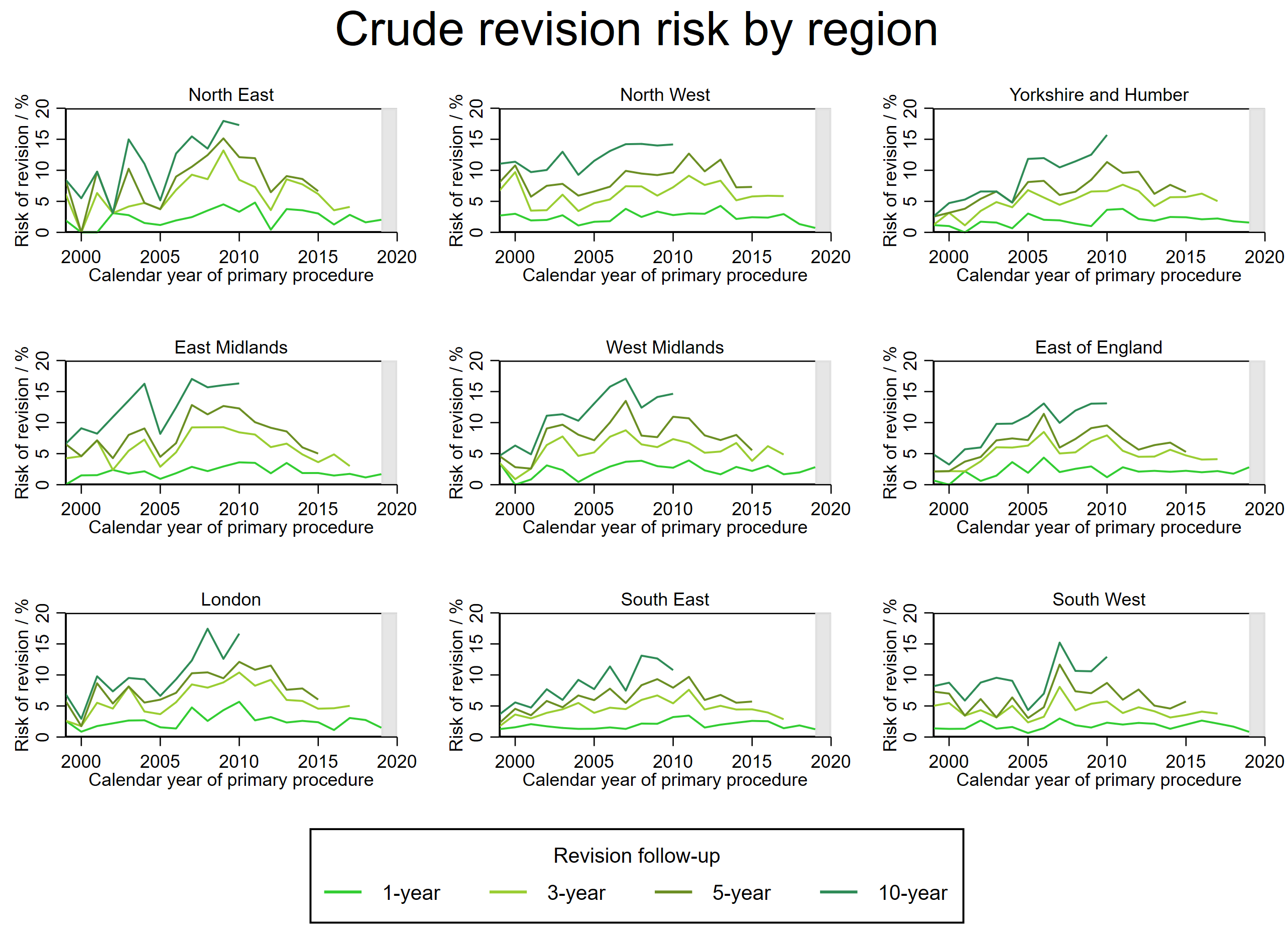


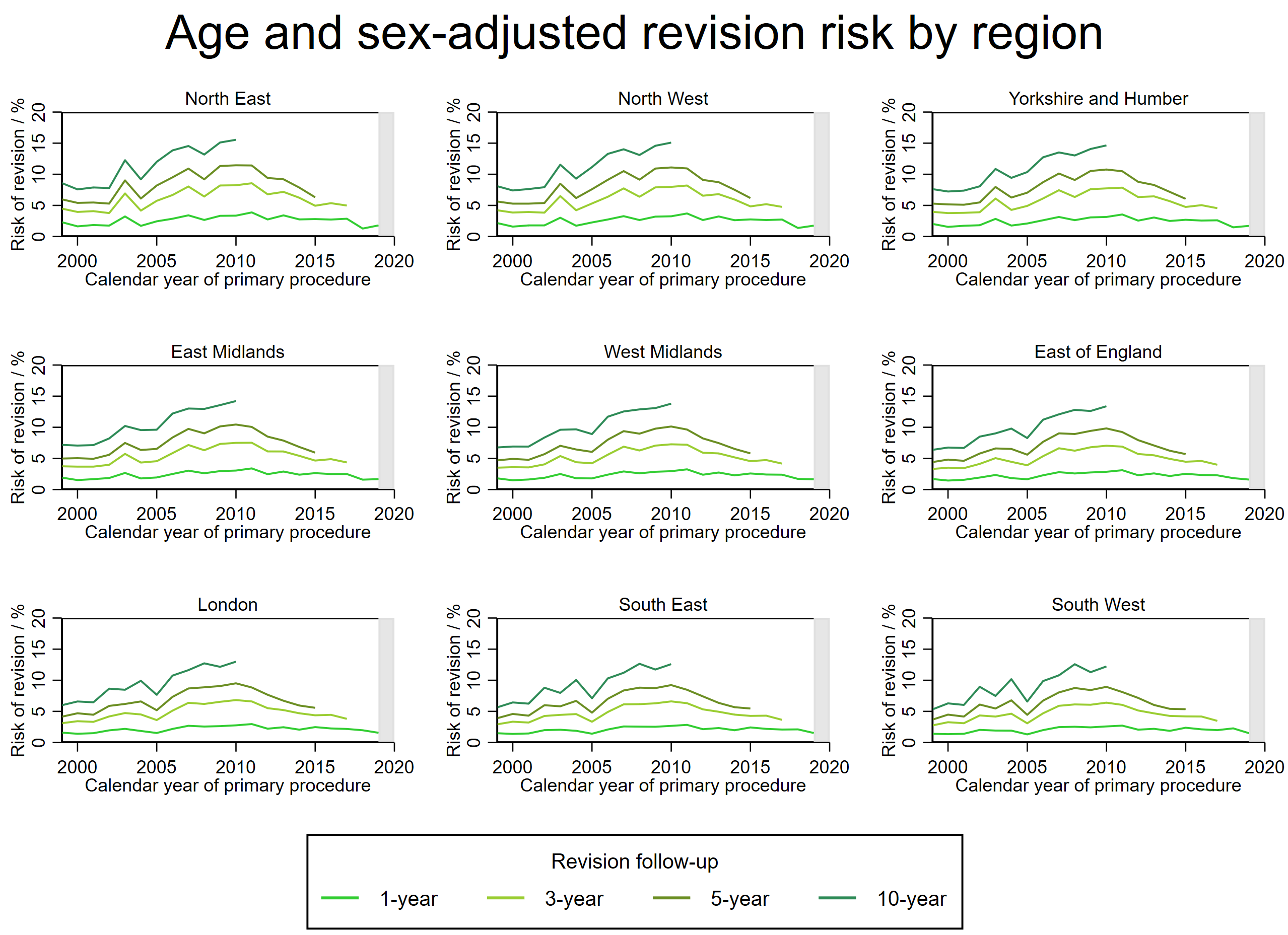


## Figure S11: Crude and adjusted revision risk by socioeconomic group


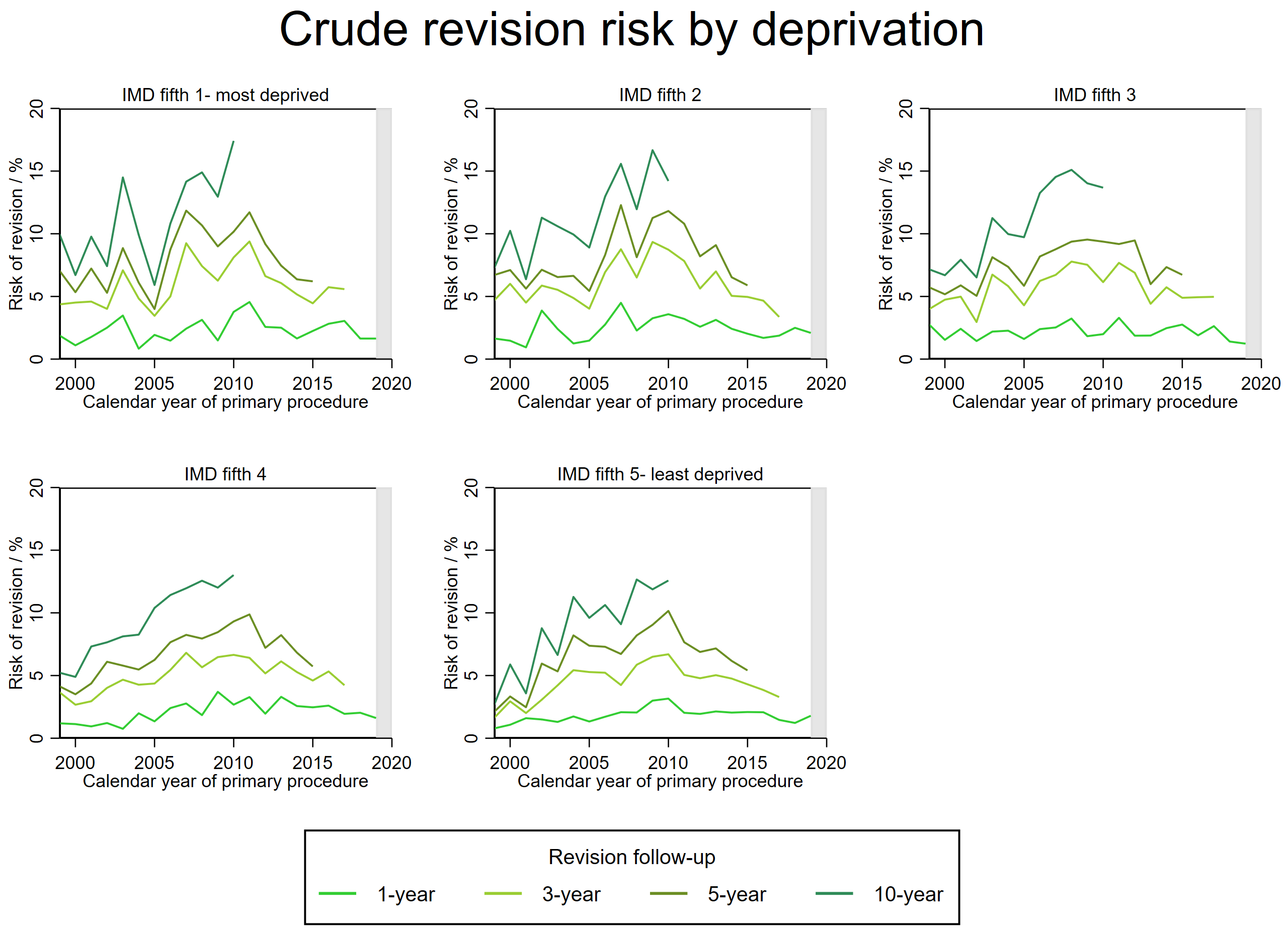


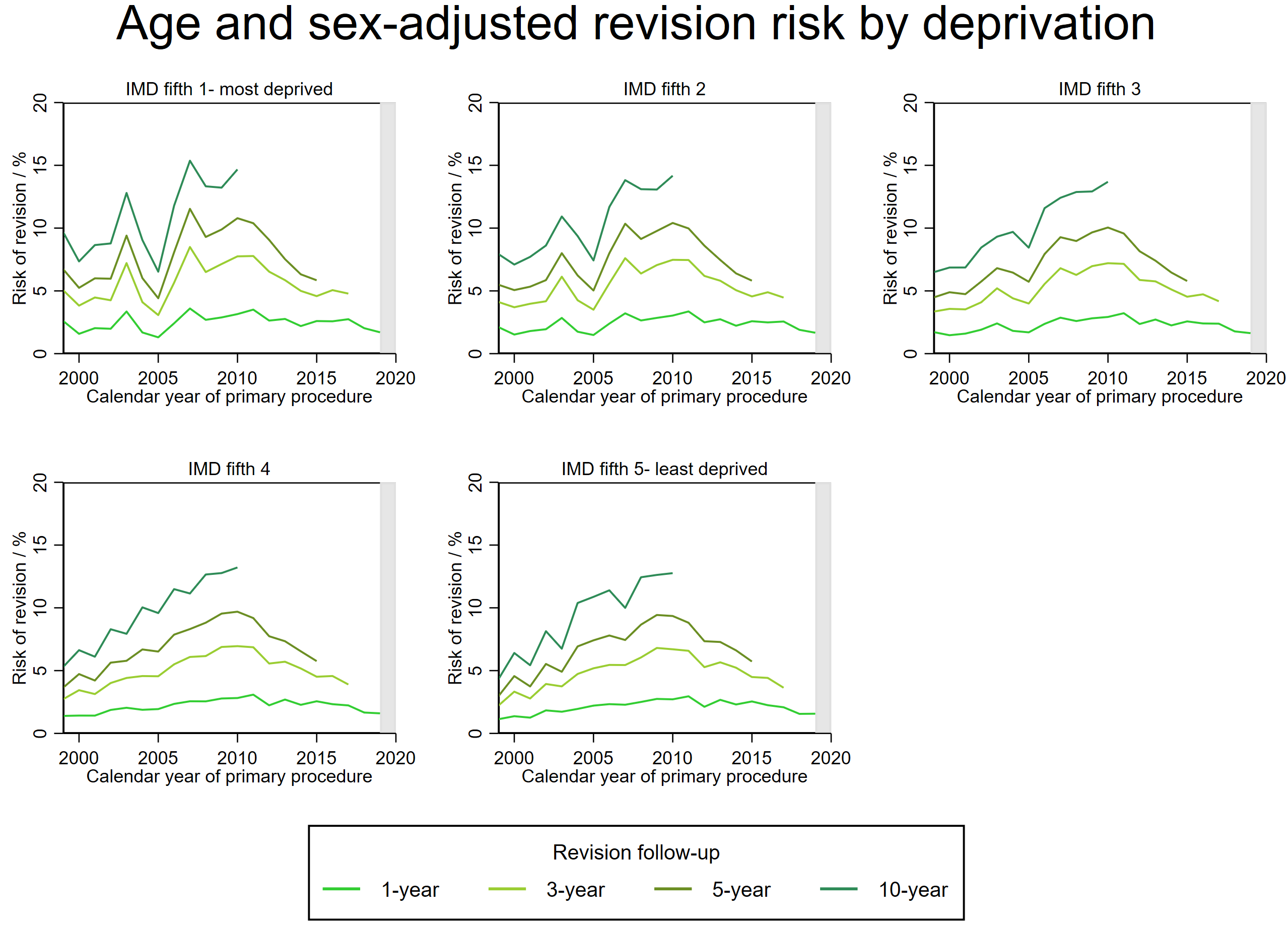


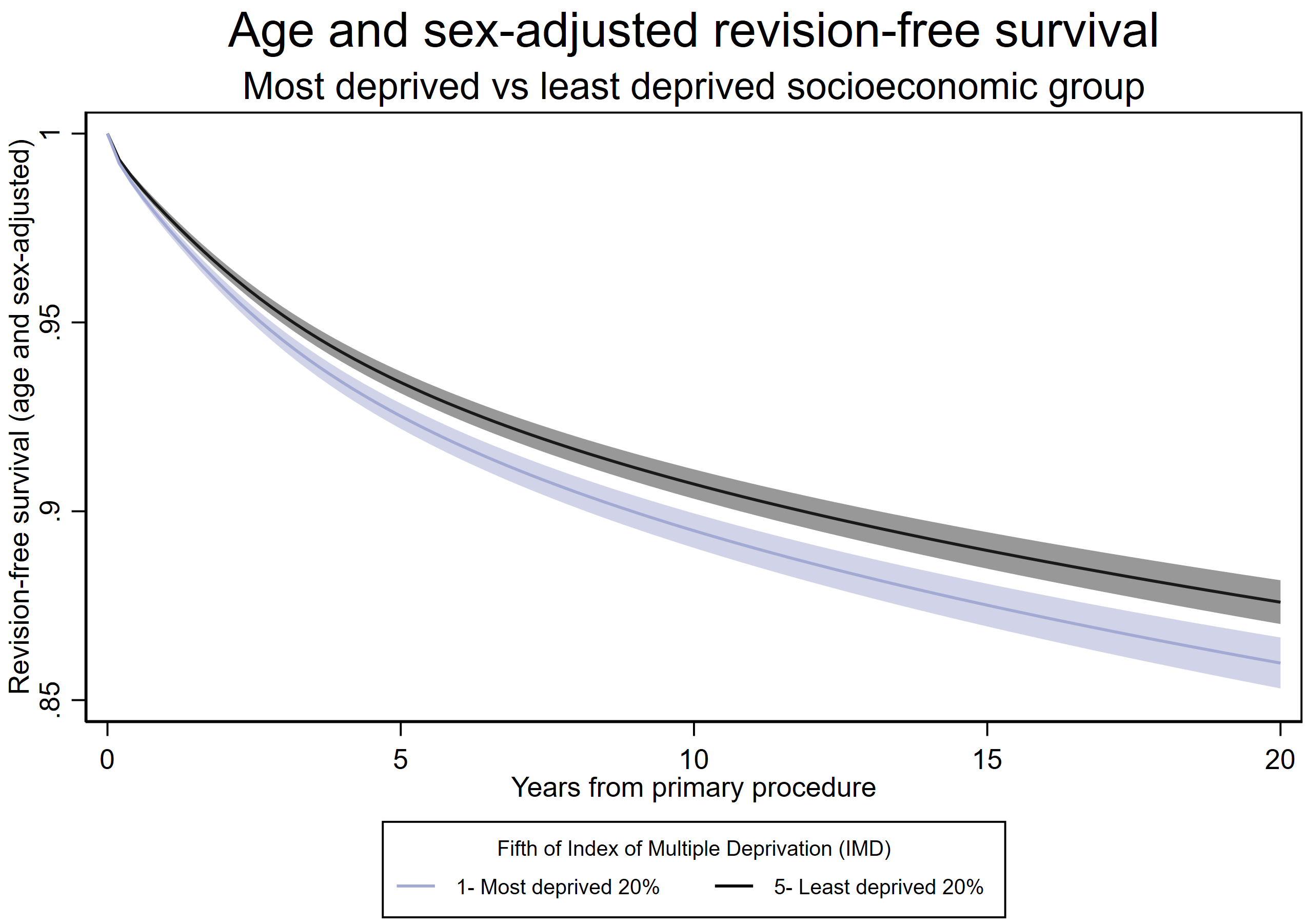


## Figure S12: Revision rates by age band


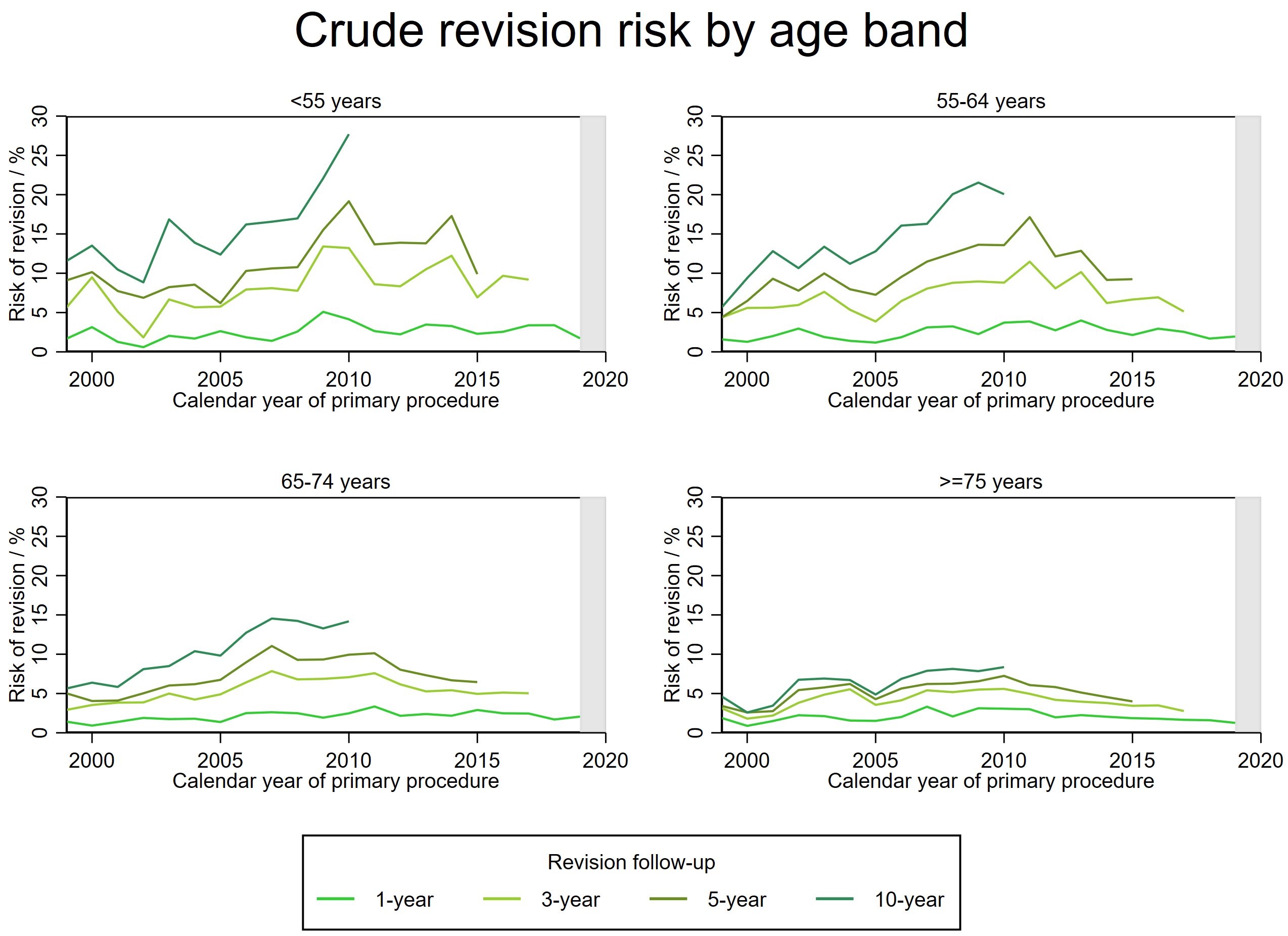


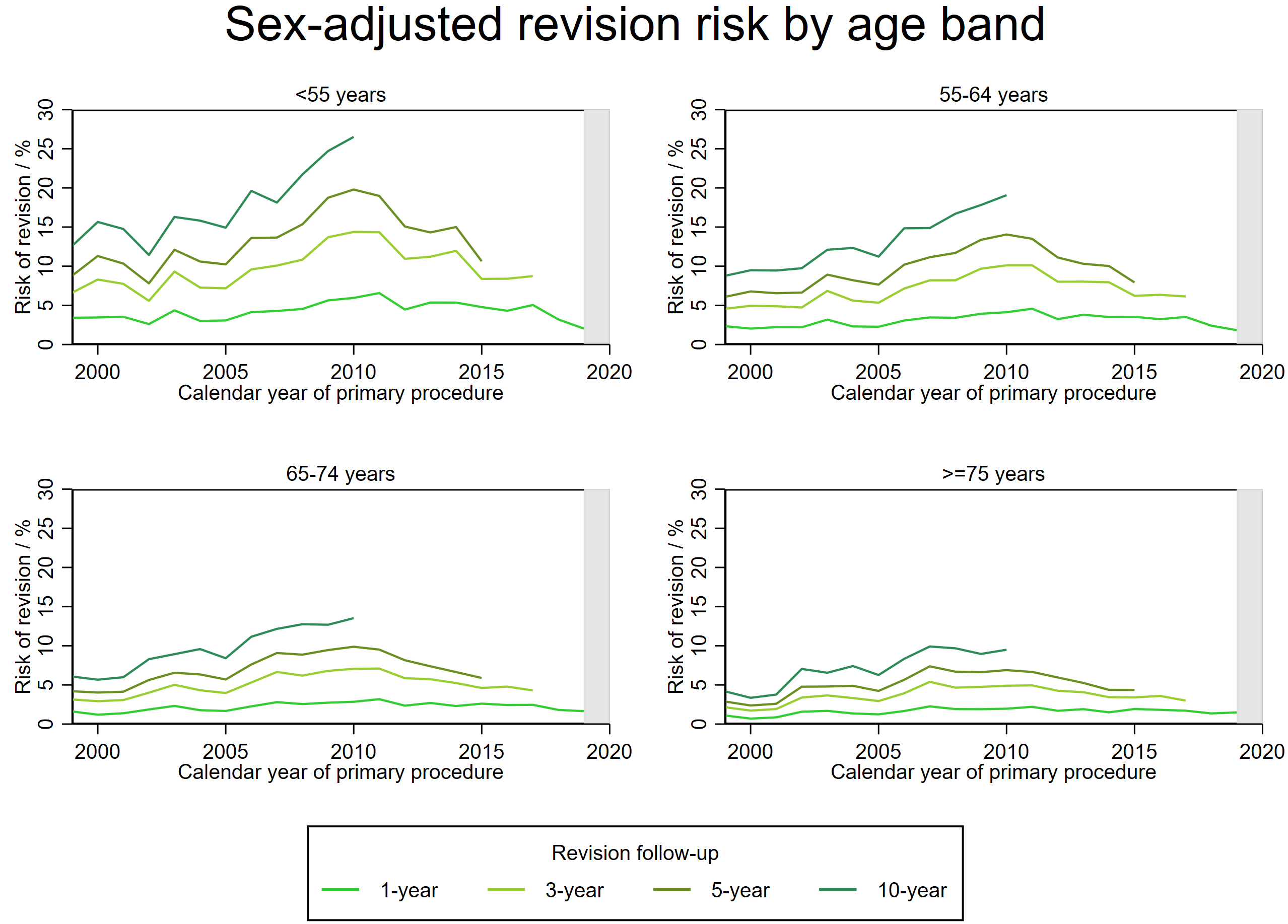


## Figure S13: Revision rates by sex


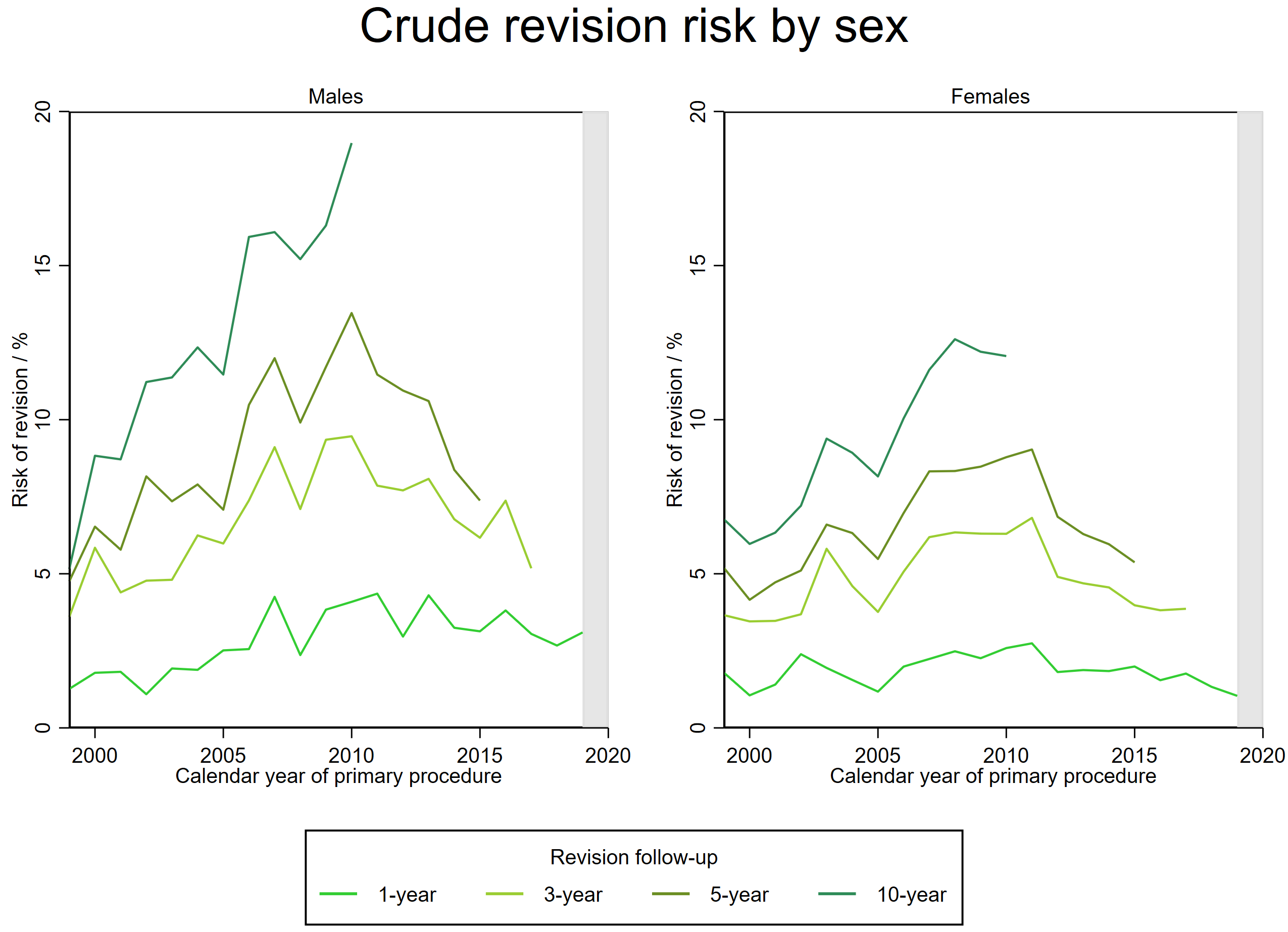


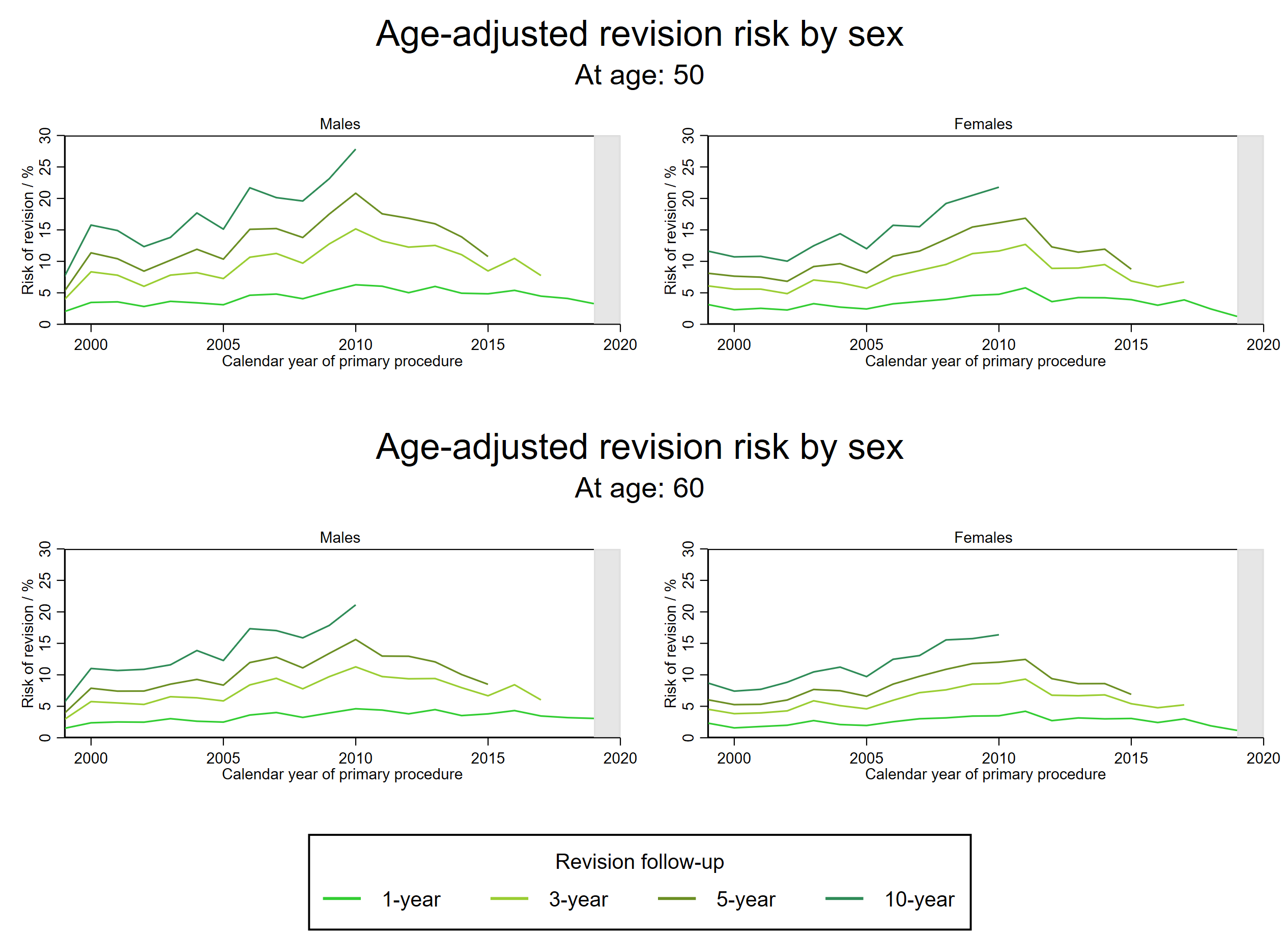


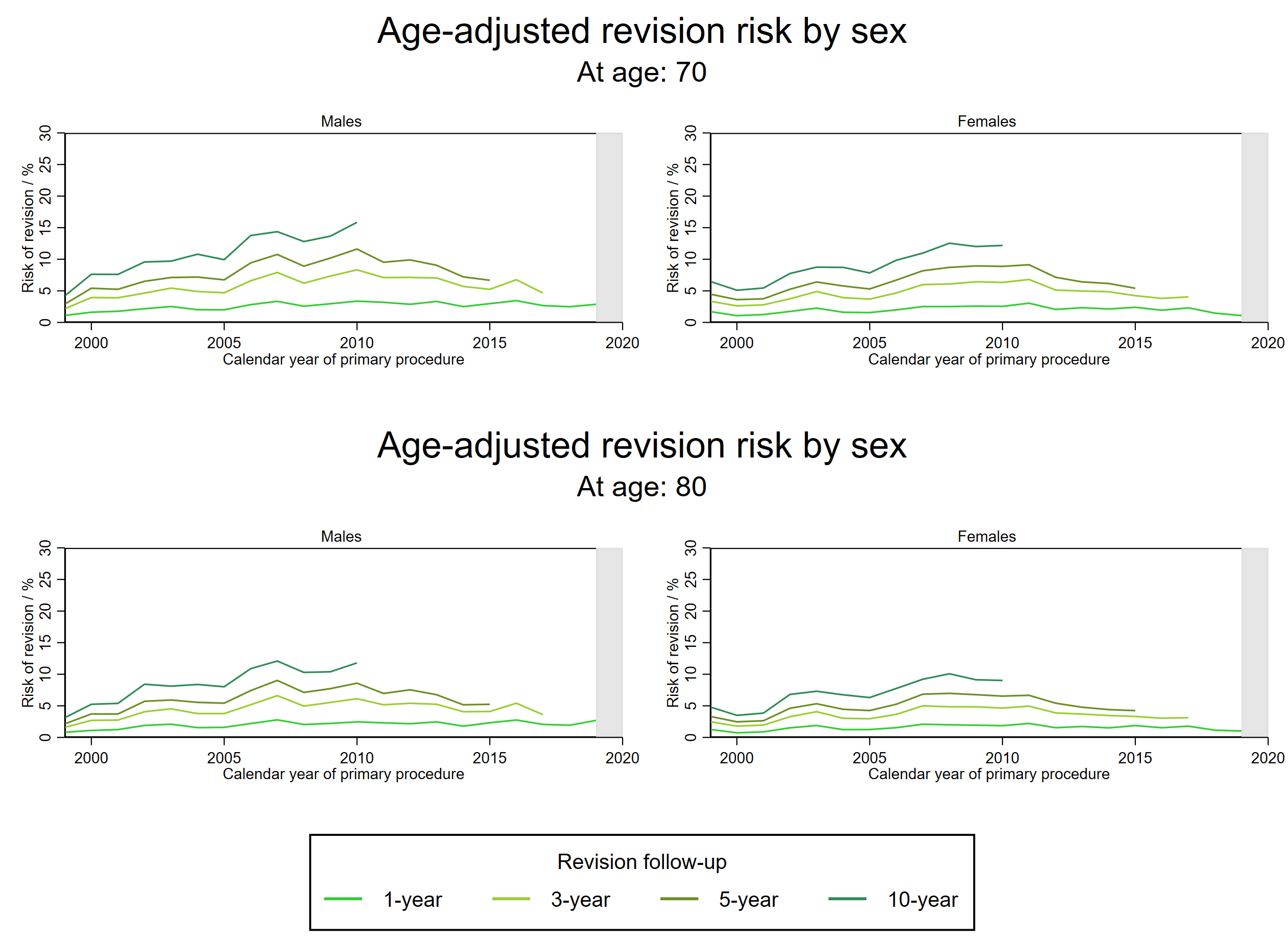

Supplement: Supplementary file 1 — Additional file 1: Table S1. HES OPCS operation codes for shoulder replacements. Table S2. HES ICD-10 codes for serious adverse events (SAE). Table S3. Patient region of treatment by region of residence. Table S4. Historic procedure counts, forecast estimates, and hospital cost. Table S6. Baseline characteristics for missing data. Table S7. Outcomes for missing data. Fig. S1. Data flow chart. Fig. S2. Average age at elective primary shoulder replacement. Fig. S3. Average age at revision shoulder replacement. Fig. S4. SAE risk by socioeconomic group. Fig. S5. SAE risk by region. Fig. S6. SAE risk by age band. Fig. S7. SAE risk by sex. Fig. S8. Breakdown of SAE risk by region. Fig. S9. Breakdown of SAE risk by socioeconomic group. Fig. S10. Crude and adjusted revision risk by region. Fig. S11. Crude and adjusted revision risk by socioeconomic group. Fig. S12. Revision rates by age band. Fig. S13. Revision rates by sex. [file 12916_2023_3112_MOESM1_ESM.docx]
